# Epigenetic modifications acetylation and deacetylation play important roles in juvenile hormone action

**DOI:** 10.1186/s12864-018-5323-4

**Published:** 2018-12-14

**Authors:** Amit Roy, Subba Reddy Palli

**Affiliations:** 1 Department of Entomology, College of Agriculture, Food and Environment, University of Kentucky, Lexington, KY 40546 USA; 2 Faculty of Forestry and Wood Sciences, EXTEMIT-K, Czech University of Life Sciences, Kamýcká 1176, Prague 6, 165 21 Suchdol, Czech Republic

**Keywords:** HAT, HDAC, Kr-h1, FOXO *Tribolium* and TcA cells

## Abstract

**Background:**

Epigenetic modifications including DNA methylation and post-translational modifications of histones are known to regulate gene expression. Antagonistic activities of histone acetyltransferases (HATs) and histone deacetylases (HDACs) mediate transcriptional reprogramming during insect development as shown in *Drosophila melanogaster* and other insects. Juvenile hormones (JH) play vital roles in the regulation of growth, development, metamorphosis, reproduction and other physiological processes. However, our current understanding of epigenetic regulation of JH action is still limited. Hence, we studied the role of CREB binding protein (CBP, contains HAT domain) and Trichostatin A (TSA, HDAC inhibitor) on JH action.

**Results:**

Exposure of *Tribolium castaneum* cells (TcA cells) to JH or TSA caused an increase in expression of *Kr-h1* (a known JH-response gene) and 31 or 698 other genes respectively. Knockdown of the gene coding for CBP caused a decrease in the expression of 456 genes including *Kr-h1*. Interestingly, the expression of several genes coding for transcription factors, nuclear receptors, P450 and fatty acid synthase family members that are known to mediate JH action were affected by CBP knockdown or TSA treatment.

**Conclusions:**

These data suggest that acetylation and deacetylation mediated by HATs and HDACs play an important role in JH action.

**Electronic supplementary material:**

The online version of this article (10.1186/s12864-018-5323-4) contains supplementary material, which is available to authorized users.

## Introduction

Immature juvenile forms of animals transform to become reproductive adults during their life cycle. In insects, two types of such transformation are generally observed; incomplete metamorphosis where nymphs turn into adults in hemimetabolous insects (e.g. true bugs, locusts & cockroaches) and complete metamorphosis which comprises dramatic morphological changes that result in the transformation from larva to the intermediate form, pupa and then to reproductive adults as observed in holometabolous insects (e.g. beetles and butterflies) [1]. Despite the differences in the strategies used by the insects, the genetic switch between different forms is tightly regulated by changes in the levels of two major hormones, ecdysteroids (20-hydroxyecdysone, 20E is the most active form) and juvenile hormones (JH) [2, 3]. 20-hydroxyecdysone works through its receptor complex, ecdysone receptor (EcR) and ultraspiracle (USP) that binds to ecdysone response elements (EcRE) present in the promoter regions of ecdysone-response genes including ecdysone-induced transcription factors (“early genes”) and others [4, 5]. Periodic pulses of 20E ensure developmental transitions during molting and metamorphosis. The presence of JH prevents metamorphosis, and the absence of JH promotes metamorphosis. Juvenile hormones function through its receptor, Methoprene-tolerant (Met) and steroid receptor coactivator (SRC, also known as FISC or Taiman). The JH/MET/SRC complex binds to JH response elements (JHRE) present in the promoter regions of JH-response genes including Krüppel homolog 1 (*Kr-h1*) and plays key roles in “status quo” action of JH [6, 7]. In *Bombyx mori*, Kr-h1 functions as a direct transcriptional repressor of *E93*, the adult specifier gene, through binding to response elements present in the promoter region of this gene [8].

In addition to signaling through nuclear receptors and transcription factors, JH may also function through membrane receptors and second messengers [9, 10]. Our previous work in *T. castaneum* has documented the dopamine D2-like receptor [G-protein-coupled-receptor (GPCR) family member] playing critical roles in regulating JH-mediated Vg uptake [11]. Cai et al., [12] reported the involvement of GPCRs during the 20E signaling suggesting that membrane receptors may be involved in this hormone signaling. Furthermore, it has been proposed that JH could regulate Ca^2+^ homeostasis within the cell during metamorphosis by interacting with membrane receptors or ion channels. Previously, farnesol (a precursor of JH) was shown to block Ca^2+^ channels [13]. It has been suggested that the JH maintains the “status quo effect” by reducing the cellular Ca^2+^ levels and when JH titers decline, such regulation is released, and the cellular Ca^2+^ levels are elevated to induce apoptosis, a process that occurs during metamorphosis [13].

In addition to metamorphosis, JH also regulates other crucial aspects of insect life including reproduction, diapause and caste differentiation [14]. In *D. melanogaster* females, JH controls yolk protein synthesis in the fat body and ovaries [15]. In males, JH regulates courtship behavior and promotes synthesis of accessory gland proteins [16–18]. The regulatory mechanisms underlying orchestrated synthesis of JH and expression of its receptors and target genes including transcription factors, co-activators for regulation of various physiological processes such as molting and metamorphosis are not yet precise.

Recent studies documented the role of CBP (or Nejire), a protein with acetyltransferase activity (HAT), as a transcriptional co-regulator in various developmental processes in *D. melanogaster* [19, 20]. In addition, the circadian rhythms in *D. melanogaster* also rely on the presence of Nejire/CBP [21]. Intriguingly, with more than 400 documented interacting partners mostly belonging to transcription factor and growth regulator families, CBP acts as a hub for transcriptional network involved in regulation of hormone signaling pathways and other fundamental physiological processes in insect life [19, 22, 23]. Recently, the involvement of CBP in the metamorphosis of *Blatella germanica* was reported [24]. The HAT activity of CBP increases acetylation of histones resulting in neutralization of lysine residues, which in turn increases accessibility of the promoters to cellular transcriptional machinery resulting in higher gene expression.

Histone deacetylases (HDACs) remove acetyl groups, increasing DNA and histone interactions resulting in repression of target gene expression through diminishing the accessibility of the promoters to transcription factors. Hence, stringent regulation of DNA packaging by HATs and HDACs can control gene expression. In insects, HDAC inhibitor activity might facilitate caste specification behavior in honey bees [25]. Epigenetic control of neural plasticity in honey bee has been documented recently [26]. Furthermore, the size and their nutrition-dependent hyper-variability of mandibles in the broad-horned flour beetle, *Gnatocerus cornutus,* are regulated by HDACs. HDAC1 RNAi in the beetle larvae caused shortening of mandibles in the adults, whereas HDAC3 RNAi resulted in hypertrophy [27]. In the case of ants, the caste-specific foraging and scouting behaviors are also regulated epigenetically by the equilibrium between CBP-mediated acetylation and HDAC-mediated deacetylation of histones in the brain [28].

However, only limited information on the contribution of HATs or HDACs to JH action is available. In the current study, we elucidated the effect of CBP and an HDAC inhibitor TSA on gene expression using TcA cells as a model system because it has already served as a robust research platform for molecular analysis of JH signaling pathway [29, 30]. The expression of CBP was knocked down by exposing TcA cells to *dsCBP*. Exposure of *CBP* knockdown cells to DMSO (control), JH or TSA followed by sequencing of RNA isolated from the treated cells revealed the influence of acetylation status of histones on the expression of genes involved in JH action.

## Materials and methods

### Cell culture

The *Tribolium castaneum* cells (BCIRL-TcA-CLG1 are gift from Dr. Cynthia Goodman from Biological control of insects research laboratory, USDA-ARS, 1503 S. Providence, Columbia, MO, USA) were grown at 28 °C in Ex-cell 420 (Sigma) supplemented with 10% Fetal Bovine Serum (Life Technologies) following the protocol described recently [31].

### Knockdown of the *CBP* gene

Double-stranded RNA targeting *CBP* gene (*dsCBP*) was synthesized using the cDNA template and MEGAscript RNAi kit (Ambion™). For knockdown experiments, TcA cells were seeded in 12-well plates. After overnight culture in the medium containing 10% FBS, the cells were treated with either 1 μg of *dsCBP* or *dsmalE* (as control dsRNA targeting a fragment of *E. coli maltase* gene) for 72 h. At the end of the incubation with dsRNA, the cells were treated with JH III or TSA dissolved in DMSO. DMSO was added to the control cells. After 6 h of hormone treatment, the TcA cells were washed with 1X PBS, harvested and processed for isolation of RNA.

Total RNA was isolated using the TRI reagent (Molecular Research Center Inc., Cincinnati, OH). The DNA was eliminated from the total RNA using DNase I (Ambion Inc., Austin, TX). RNA quantity was measured using a NanoDrop 2000 spectrophotometer (Thermo Fisher Scientific), and quality was determined by running on 1% agarose gel. Two micrograms of total RNA from each sample was used for cDNA synthesis. cDNA was used to amplify a fragment of the target gene, and the PCR product was used for dsRNA synthesis. Primers used for dsRNA synthesis and reverse-transcriptase quantitative real-time PCR (RT-qPCR) are listed in Additional file 1: Table S1 or reported in our previous publication [32]. The MEGAscript RNAi Kit (Ambion Inc., Austin, TX) was employed for dsRNA synthesis. After total RNA extraction, cDNA synthesis and RT-qPCR were performed following the protocol described previously [33].

### RNA-seq study design, library preparation, and sequencing

Total RNA was extracted from *dsmalE* (M) treated cells exposed to DMSO (*dsmalE*+ DMSO or MD), JH III (*dsmalE*+ JHIII or MJ) or TSA (*dsmalE*+ TSA or MT) and dsCBP (C) treated cells exposed to DMSO (*dsCBP*+ DMSO or CD), JH III (*dsCBP*+ JHIII or CJ) or TSA (*dsCBP*+ TSA or CT). Three biological replicates were used for each treatment. The design of RNA seq experiment is presented in Table 1. The protocol used for RNA seq library preparation include oligo-dT based total RNA separation, RNA fragmentation, two-step cDNA preparation and library barcoding, as described previously [33, 34]. Briefly, mRNA was purified and enriched from 3 μg of high-quality RNA from each biological replicate, fragmented and used for cDNA preparation. First strand cDNA synthesis was performed using SMARTSCribe reverse transcriptase enzyme (Clontech Laboratories, Inc) and reverse transcriptase primers (Integrated DNA technologies) with a sequencing adaptor followed by a random hexamer for Illumina sequencing and unique barcode for multiplexing. Second strand cDNA was synthesized using SMART 7.5 primer (Clontech Laboratories, Inc). The cDNA fragments were size selected (~ 300 bp) using HighPrep TM PCR beads (MAGBIO) and finally amplified using PCR. Libraries are quantified by Nanodrop and verified by running them on 1.2% agarose gels. Finally, the libraries were multiplexed and sent for sequencing (single-end) using the HiSeq4000 sequencer at the Genomics Technologies Center of Duke University, NC, USA. Raw sequencing data statistics are presented in Table 2.

**Table 1:** RNA-seq study design

No	Comparisons	Abbreviation
1	DMSO and JH treated cells exposed to dsmalE	MD vs MJ
2	DMSO treated cells exposed to dsmalE or dsCBP	MD vs CD
3	DMSO or JH treated cells exposed to dsmalE or dsCBP	MD vs CJ
4	DMSO or TSA treated cells exposed to dsmalE or dsCBP	MD vs CT
5	DMSO or TSA treated cell exposed to dsmalE	MD vs MT

The table shows a list of comparisons made after RNA sequencing. All the treatments are compared with the control (MD)

**Table 2:** RNA-seq statistics

	dsmalE			dsCBP		
Samples	MD1-MD3	MJ1-MJ3	MT1-MT3	CD1-CD3	CJ1-CJ3	CT1-CT3
Total no. of reads	90,280,120	64,052,276	86,015,875	98,406,728	100,327,204	94,045,683
No. of high-quality reads	80,437,710	57,466,708	54,928,048	86,047,960	79,067,179	79,188,207
Mapped reads to Exon^a^	75,27,476	53,578,082	48,830,575	81,330,901	73,271,933	71,579,430
Percent reads mapped	83.59	93.29	87.89	93.36	92.97	90.37
Uniquely mapped reads^b^	70,309,618	49,895,642	45,547,725	75,753,713	68,994,581	67,063,490

^a^Exon regions of *Tribolium castaneum* genome

^b^Reads considered for downstream gene expression analysis

The table is showing a summary of read statistics after Illumina Hi-seq 4000 sequencing. Total read counts for three biological replicates from each treatment varies within 64,000,000 to 101,000,000. Number of high quality reads indicates the number of reads left after read trimming and filtering (Quality control step). Percentage of high quality reads unambiguously mapped to reference *Tribolium* genome represented in the second last row. Uniquely mapped reads were considered for differential gene expression analysis. Further details of RNA-seq statistics per sample are given in Additional file 1: Table S2

### RNA-seq data analysis

Raw reads after quality control (demultiplexing, trimming and adaptor removal) were mapped to the reference genome (GA-2 strain of *T. castaneum*) [35]. The parameters used for the mapping are mismatch cost = 2, insertion cost = 3, deletion cost =3, length fraction = 0.8, similarity fraction = 0.8, unique exon read mapping. The read counts were Log_10_ transformed followed by normalization using the scaling method where the sets of the transformed expression values for the samples were multiplied by a constant so that the sets of normalized values for the samples had the same ‘target’ value. The RNA-seq tool from CLC workbench (Version 9.5.9) was used to quantify gene expression employing standard pre-optimized settings and parameters such as mapping to exon regions only [36]. Further, biases in the sequence datasets and different transcript sizes were corrected using the RPKM algorithm to obtain correct estimates for relative expression levels. Finally, EDGE (Empirical analysis of differential gene expression) analysis was performed through “Advanced RNA-Seq plug-in” in CLC workbench using the recommended parameters such as estimated count filter cut off 5, estimate tag-wise dispersion [33]. For differential gene expression, we set a *p*-value cut-off of < 0.01 and fold change cut-off of ±2-fold as a threshold value for being significant. Differentially expressed genes were functionally annotated using “cloud blast” feature within the “Blasto2GO plug In” in CLC Genomic Workbench (Version 9.5.9). Nucleotide blast was performed against arthropod database with an E-value cut off 1.0E-3. Both, annex and GO slim were used to improve the GO term identification further by crossing the three GO categories (biological process, molecular function, and cellular component) to search for name similarities, GO term and enzyme relationships within KEGG (Kyoto Encyclopedia of Genes and Genomes) pathways.

### RT-qPCR validation

RT-qPCR was conducted to validate the RNA seq results with a subset of 15 genes induced after JH treatment. Primers were designed using IDT’s primer design software (www.idtdna.com) with parameters including length 18–25 nt, melting temperature 55–65 °C, GC content 50–60% and product size 100–150 bp (Additional file 1: Table S1). Primers were tested by PCR followed by gel electrophoresis for correct product amplification. cDNA for RT-qPCR were synthesized using RNA from a new set of dsmalE, and dsCBP treated samples. cDNA was prepared from 3 μg RNA per sample using M-MLV reverse transcriptase kit (Invitrogen TM) following the manufacturer protocol. Resulting cDNA samples were diluted (4 times), and RT-qPCR was performed using SYBR Green Supermix (Bio-rad) following existing lab protocol [33]. Melt curves were generated to ensure single product amplification. The target genes expression levels were calculated using the 2-ΔCt method with RP49 (a ribosomal protein) as a reference gene, which had already been validated as a housekeeping gene in *Tribolium castaneum* [37–39].

## Results and discussion

Ecdysteroids and JH regulate insect metamorphosis by performing antagonistic roles [40]. 20E facilitates metamorphosis whereas the presence of JH prevents it until the appropriate time. The role of JH in preventing precocious metamorphosis is documented in some hemimetabolous and holometabolous insects [6, 41–43]. We studied the contribution of CBP (HAT, acetylation) and TSA (HDAC inhibitor) to JH action. TcA cell line, originated from the non-embryonic adult insect tissues, was used in the study. The cells were treated with dsmalE (dsRNA targeting *E. coli maltase* gene as control) or *dsCBP* followed by exposure to JH III, TSA or DMSO (Table 1).

### TcA cell response to different treatments

**Fig. 1:**
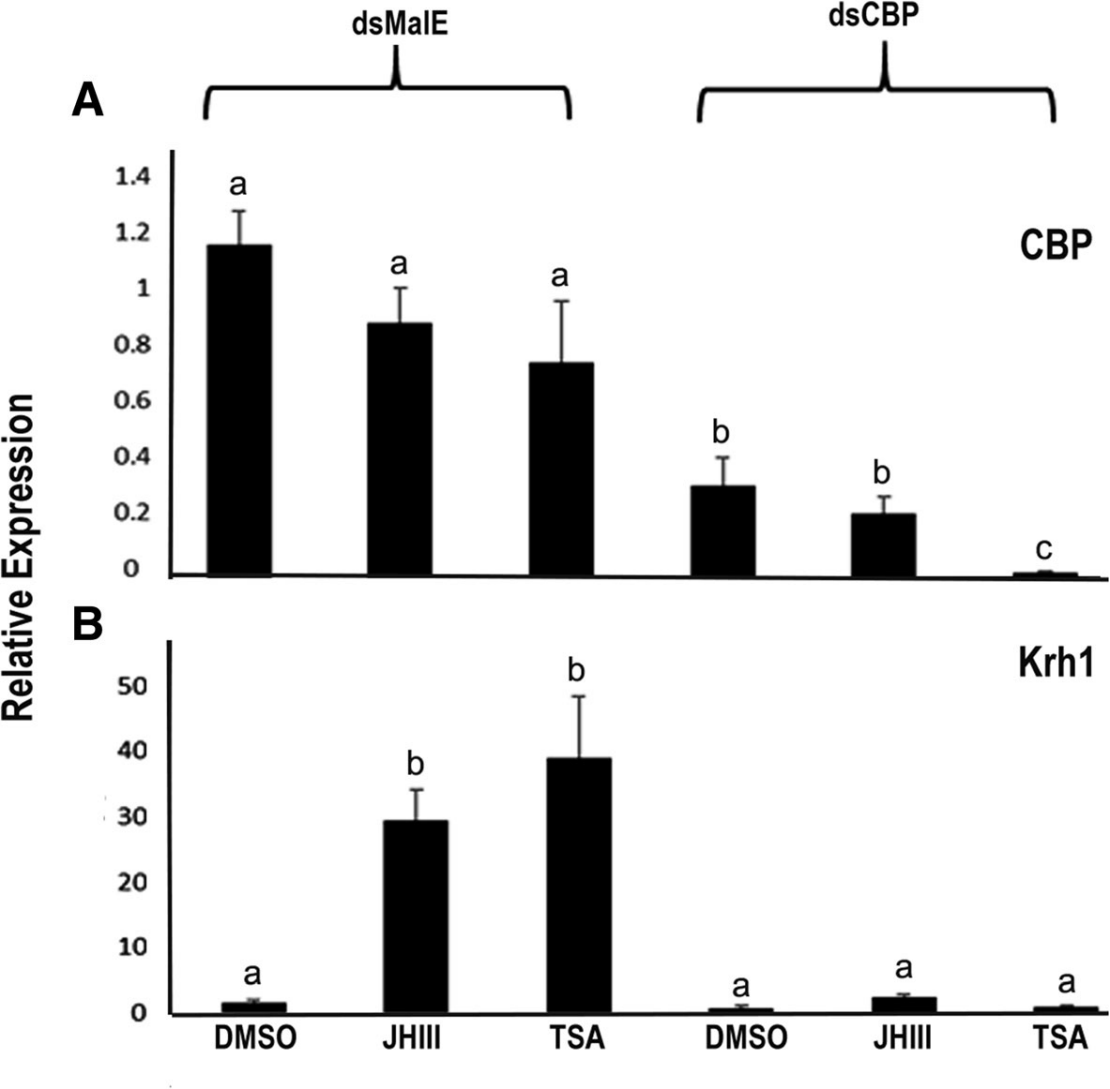
TcA cells respond to both juvenile hormone and TSA after dsmalE and dsCBP treatment. Total RNA was isolated from 100,000 cells that were cultured in the medium containing either dsmalE or dsCBP for 72 h. The cells were then exposed to DMSO (< 0.1%) or JH III (2 μM) or TSA (5 μM) for 6 h. Total RNA was isolated from the treated cells and used to quantify mRNA levels of CBP (**a**) and Kr-h1 (**b**). The data shown are the mean + S.E. (*n* = 4). (Letters represent significance at 95% CI)

The response of TcA cells to different treatments [MD (*dsmalE*+ DMSO), MJ (*dsmalE*+ JHIII), MT (*dsmalE*+ TSA), CD (*dsCBP*+ DMSO), CJ (*dsCBP*+ JHIII), and CT(*dsCBP*+ TSA)] was determined before the sequencing experiments by quantifying the expression of *CBP* and *Kr-h1* genes in all the treatments using RT-qPCR method [33]. Results showed more than 50% reduction of *CBP* mRNA levels after *dsCBP* treatment. The *Kr-h1* mRNA levels in control cells exposed to dsmalE increased after JH or TSA treatment when compared to their levels in DMSO treated cells. However, there was no increase in *Kr-h1* mRNA in cells exposed to dsCBP and treated with JH or TSA indicating the requirement of *CBP* for the cellular response to JH and TSA treatments (Fig. 1). These results suggest a possible role for *CBP* and TSA in JH induction of *Kr-h1* gene.

### RNA-seq and gene expression analysis

**Fig. 2:**
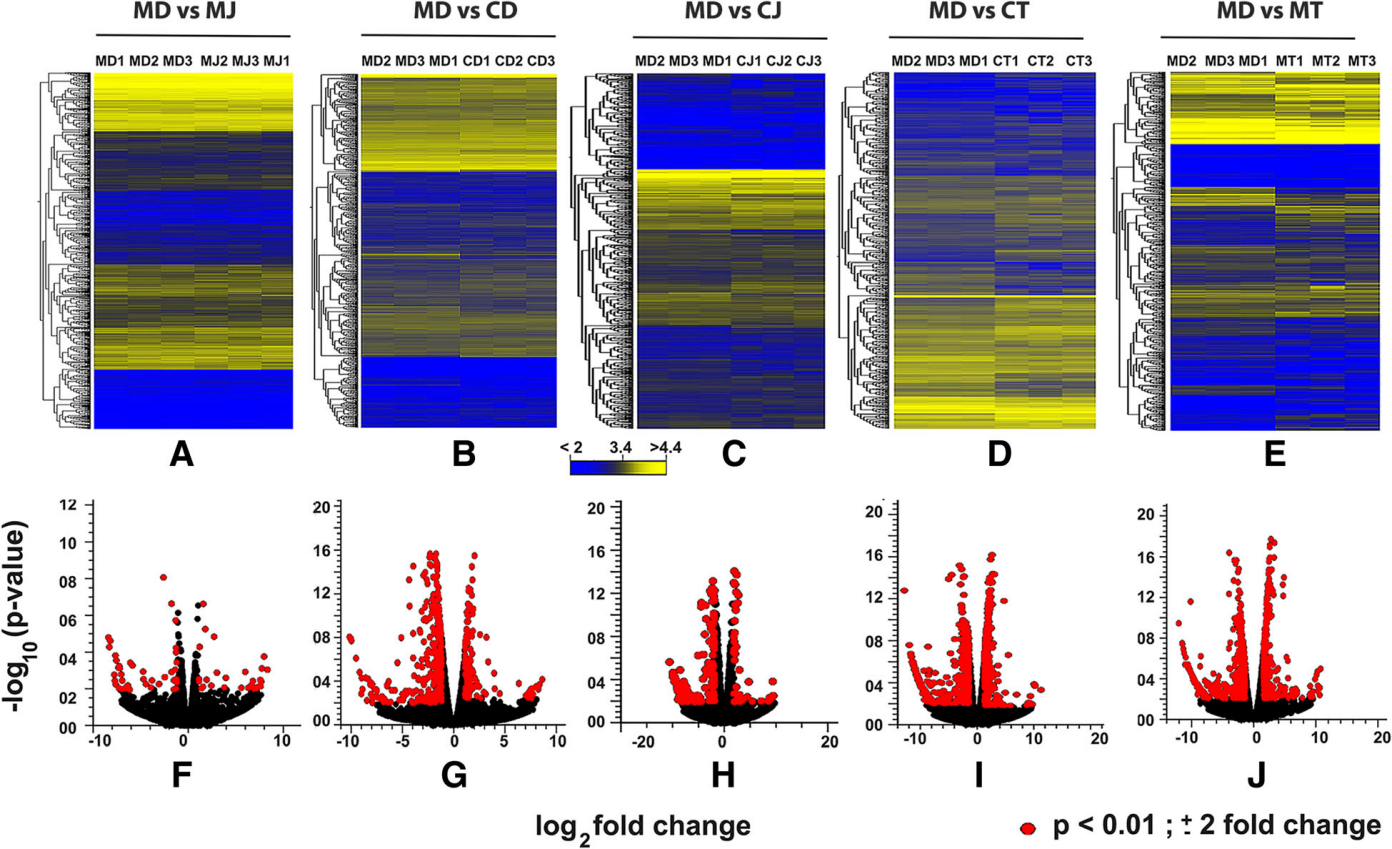
Gene expression comparisons in TcA cells exposed to dsmalE or dsCBP and treated with JH III and TSA using the empirical analysis of DGE (EDGE) algorithm in CLC Genomics Workbench (Version: 9.5.9). (**a**-**e**) Heatmaps showing the overall comparison between DMSO and JH III treated cells exposed to dsmalE (MD vs. MJ), DMSO treated cells exposed to dsmalE or dsCBP(MC vs. CD), DMSO or JH III treated cells exposed to dsmalE or dsCBP(MD vs. CJ), DMSO or TSA treated cells exposed to dsmalE or dsCBP (MD vs CT), and DMSO or TSA treated cells exposed to dsmalE (MD vs. MT) (Table 1). The dsmalE plus DMSO treated cells act as a control. The color spectrum, stretching from yellow to blue, represents TMM (trimmed mean of M values) normalized expression values obtained after EDGE analysis. (**f**-**j**) Volcano plots of expression data after EDGE analysis. The red dots indicate the number of significantly up- and down-regulated genes using *p* < 0.01 and ± 2-fold change as the cut-off threshold corresponding to each comparison

To identify the impact of *CBP* on gene expression after JH III or TSA treatment, we performed sequencing of RNA isolated from TcA cells exposed to *dsmalE* or *dsCBP* followed by treatment with JH or TSA. Illumina sequencing resulted in 90.28, 64.05, 86.01, 98.4, 100.32 and 94.04 million reads for MD (*dsmalE*+ DMSO), MJ (*dsmalE*+ JHIII), MT (*dsmalE*+ TSA), CD (*dsCBP*+ DMSO), CJ (*dsCBP*+ JHIII) and CT (*dsCBP*+ TSA) treatments respectively. After quality control measures, approximately 83–93% of reads from all treatments were mapped back to the exon regions of the *T. castaneum* genome (Table 2). Further details of RNA-seq statistics for each biological replicate are included in Additional file 1: Table S2. After RPKM based gene expression normalization, differential gene expression (DGE) analysis was performed using EDGE analysis software package of CLC workbench, and the overall gene expression patterns for each treatment compared to the control (MD) are represented as heatmaps (Fig. 2a-e). Comparing with the control (MD), the gene expression levels in different samples was normalized in a way that made all treatments comparable to each other. Differentially expressed (*p* < 0.01 and ± 2-fold ↑↓) genes for each comparison (Table 1) are represented as red dots in the volcano plots (Fig. 2f-j).

**Fig. 3:**
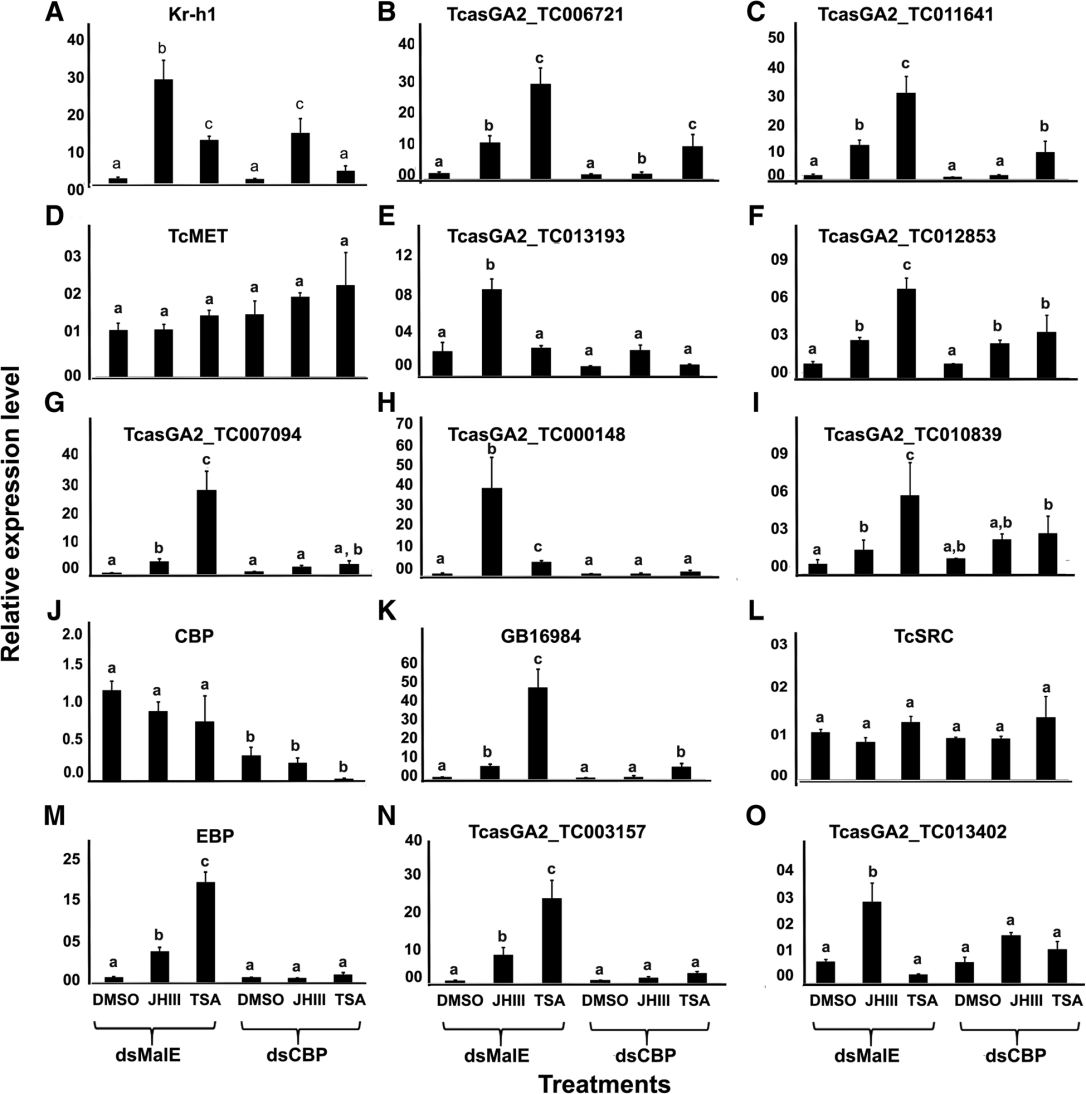
Relative mRNA levels of a subset of 15 genes selected from the genes that were upregulated after JH III application in different treatments. Relative mRNA levels of these genes were determined by RT-qPCR as described in Fig. 1. The data shown are the mean + S.E. (n = 4). (Letters represent significance at 95% CI). [TcasGA2_TC006721: myb-like protein X; TcasGA2_TC011641: alpha-2C adrenergic receptor; TcasGA2_TC013193: juvenile hormone esterase; TcasGA2_TC012853: Mmp-3; TcasGA2_ TC007094: TBC1 domain family member 19; TcasGA2 _TC000148: CYP345C1; TcasGA2_ TC010839: Vitellogenin; GB16984: nicotinic acetylcholine receptor alpha9 subunit (nAChRa9); TcasGA2_TC003157: uncharacterized LOC103312232; TcasGA2_TC013402: aromatic-L-amino-acid decarboxylase]

After statistical analysis, 32 and 699 genes were upregulated after JH (MD vs. MJ) or TSA (MD vs. MT) treatment of control cells exposed to *dsmalE*. Similarly, 181 and 602 genes were significantly upregulated in TcA cells exposed to *dsCBP* followed by treatment with JH (MD vs. CJ) or TSA (MD vs. CT). Also, *CBP* knockdown alone caused down-regulation of 456 genes in DMSO treated cells compared to control (MD vs. CD). Details on Blast2GO hits in the NR databases are included in Additional files 2, 3, 4, 5 and 6: Excel Files S1-S5. The expression of 15 selected genes from all the treatments was verified by RT-qPCR (Fig. 3). Comparison of the gene expression levels between RT-qPCR and RNA-seq study showed a correlation coefficient of 0.74 (Additional file 1: Figure S1) indicating a good agreement of gene expression data obtained by both methods.

### JH response genes in TcA cells

**Fig. 4:**
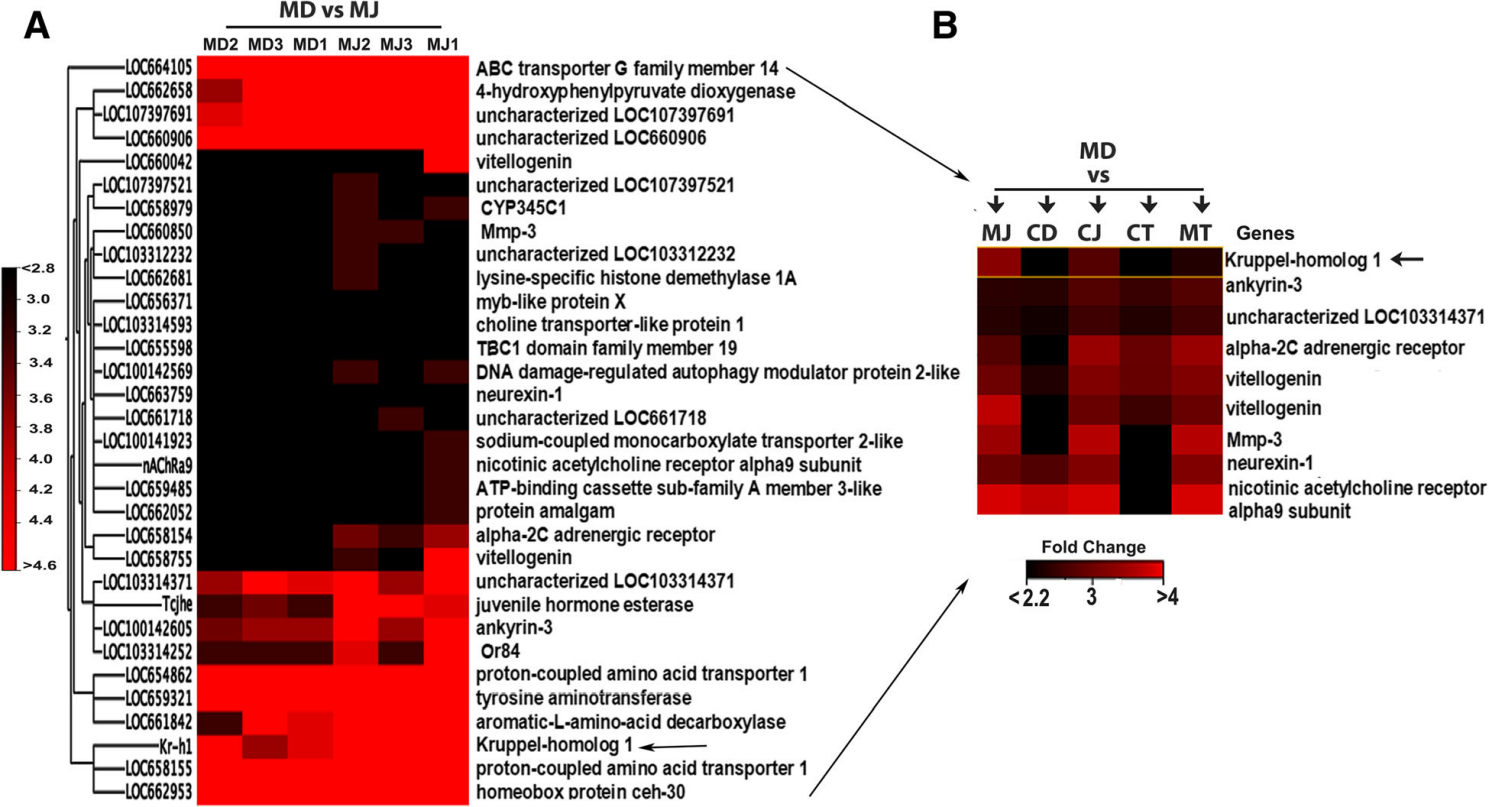
Gene expression after JH III induction. (**a**) Heatmap illustrating the TMM normalized expression values of 32 up-regulated genes (*p* < 0.01, ≤ 2-fold↑) after dsmalE and JH III treatment in TcA cells. (**b**) Heatmap showing the expression of genes in different treatments that are behaving similarly to Kr-h1, the major JH-response gene. [MD: dsmalE (M) treated cells exposed to DMSO; MJ: dsmalE (M) treated cells exposed to JH III]

JH III treatment of TcA cells (*dsmalE*+ JHIII or MJ) caused an increase in expression of known JH response genes including *Kr-h1* (Fig. 4a), a mediator of anti-metamorphic action of JH [44–47] when compared to control cells treated with DMSO (MD or *dsmalE*+ DMSO). A decrease in JH titers at the end of final instar larval stage reduces the *Kr-h1* mRNA levels that relax the inhibitory influence on metamorphic changes and leads to the onset of metamorphosis through an increase in expression of genes such as *Broad* and *E93* required for pupal and adult development respectively [8, 48]. In *T. castaneum*, RNAi-mediated depletion of *Kr-h1* expression led to precocious pupation [45]. Besides *Kr-h1*, there were 31 other genes whose expression increased after JH treatment of TcA cells. Among them, eight genes including *ankyrin 3*, *Vitellogenin* (*Vg*), *matrix metalloproteinase-3* (*Mmp-3*), *neurexin-1* and *alpha-2C adrenergic* receptor showed expression pattern similar to *Kr-h1* in different comparisons (Fig. 4b). Ankyrin or ankyrin repeat containing proteins are involved in signal transduction processes in *D. melanogaster* and play a crucial role in the development or initiation of the immune response in eukaryotic cells [49–51]. Induction of *ankyrin-3* gene by JH suggests its function in the regulation of gene expression in response to the immune challenge as reported previously [52]. Induction of Vg (a protein made by female insects to produce yolk) by JH in TcA cells support our previous finding on the regulation of *Vg* gene in *T. castaneum* by JH through insulin-like peptide signaling pathway [53]. In other insects including *B. germanica*, ectopic application of JH caused re-expression of *Vg* in JH-deficient female adults [54]. Similarly, in *Locusta migratoria*, induction of *Vg* was observed after methoprene (JH analog) injection [55].

Furthermore, JH also induced *Mmp-3*, and it showed a similar expression profile as *Kr-h1* after various treatments. In *T. castaneum*, three Mmps, i.e., *Mmp1*, *Mmp2*, *and Mmp3* were identified [56, 57]. RNAi mediated knockdown of *Mmp1* caused an arrest in the initial stage of pupation resulting in the altered development of wings, legs, antennae, compound eye, etc. Whereas, *Mmp2* knockdown resulted in abnormal gut development during beetle embryogenesis [58]. However, RNAi mediated knockdown of *Mmp3* did not show any abnormality although the expression of *Mmp3* was highly upregulated during metamorphosis. Hence, it was postulated that both *Mmp2* and *Mmp3* had overlapping functions. Moreover, JH also induced an *alpha 2C adrenergic receptor*, a G-protein coupled receptor (GPCR) in TcA cells. Interaction of GPCR with JH had been reported in other insects [9, 59]. Our previous studies in *T. castaneum* screened multiple candidate GPCRs that may be involved in Vg uptake into oocytes and identified promising candidate membrane receptor mediating JH regulation of patency [11]. However, the contribution of intracellular and membrane receptors in JH action need to be investigated further.

### TSA-response genes

**Fig. 5:**
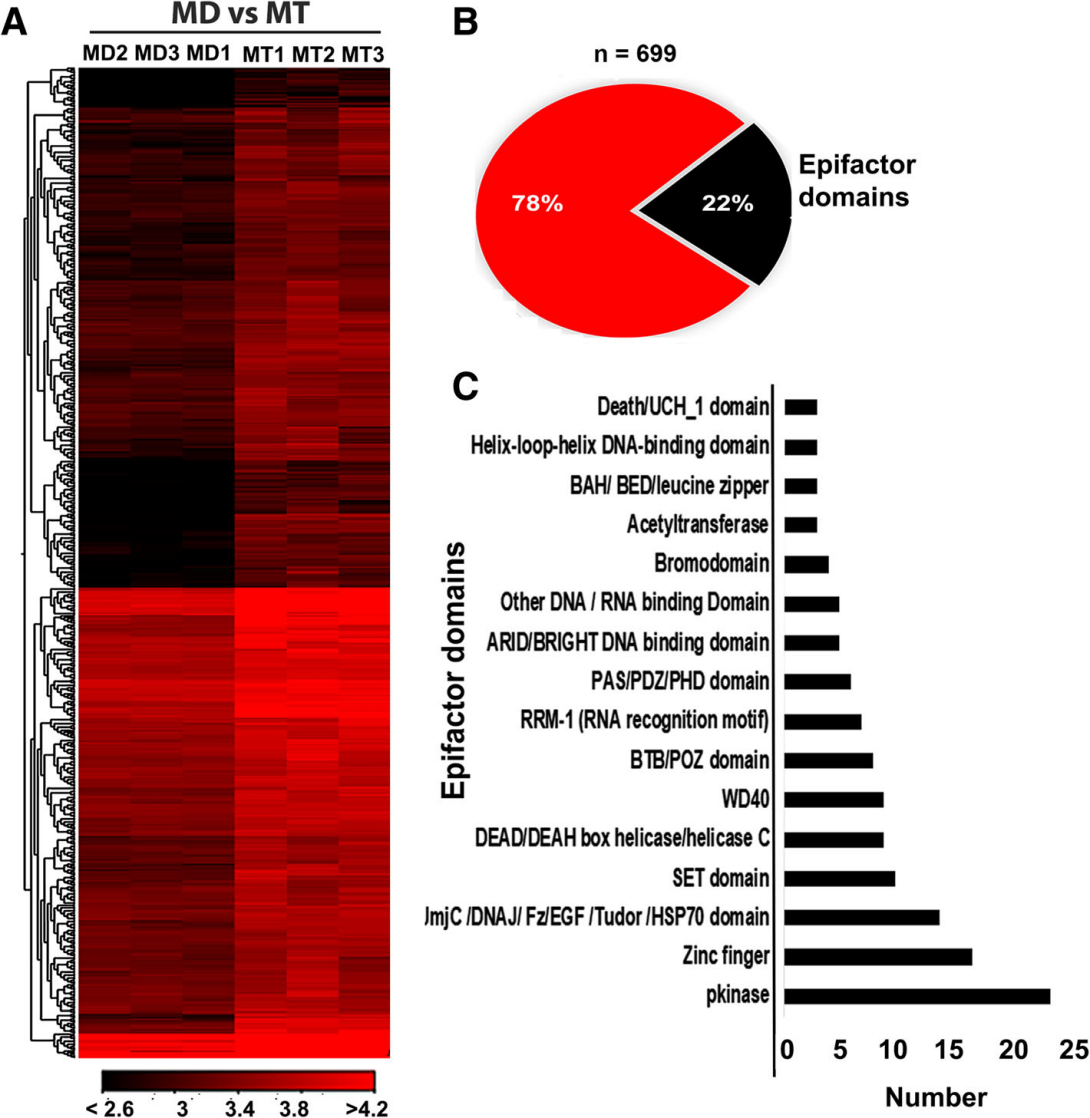
Effect of TSA application on gene expression. (**a**) Heatmap illustrating the TMM normalized expression values of 699 up-regulated genes (*p* < 0.01, ≥ 2-fold↑) after TSA treatment in TcA cells. (**b**) Figure showing the percent containing epifactor domains within 699 genes. (**c**) Epifactor domains present in TSA induced genes. [MD: dsmalE treated cells exposed to DMSO; MT: dsmalE treated cells exposed to TSA]

Trichostatin A (TSA) is a class I and II histone deacetylase (HDACs) inhibitor. It is known that HDACs mediated epigenetic mechanisms regulate various physiological processes [60]. In the present study, TSA application resulted in up-regulation of approximately 699 genes in dsmalE treated cells (MT or *dsmalE* + TSA) when compared to control cells treated with DMSO (MD or *dsmalE* + DMSO) (Fig. 5a, Additional file 3: Excel File S2). 22% of these genes contained “epifactor” domains (Fig. 5b) that are detected most frequently in proteins with capabilities of modifying histones and thus involved in chromatin remodeling [61]. Epifactor domains, observed predominantly within the gene set induced by TSA are shown in Fig. 5c. Some of these domains are well known due to their involvement in various cellular processes. For example, the SET domain proteins are involved in DNA methylation [62], and the Jmj (Jumonji) domain proteins are involved in histone demethylation [63]. It is known that ‘eggless’ family of SET domains fused to the TAM/MBD domain play a distinct role in epigenetic regulation through DNA and histone methylation in *Acyrthosiphon pisum* [64]. In MD vs. MT comparison, we identified nine SET domain-containing genes that are induced by TSA (Fig. 5c & Additional file 3: Excel File S2). Moreover, genes coding for polycomb group protein Psc and trithorax that maintain active or repressed gene expression states in a cell by regulating chromatin architecture at multiple levels ranging from local structural modifications to the three-dimensional organization within the genome were also induced by TSA treatment. Indeed, most prominent epigenetic regulatory systems use these well-conserved groups of proteins to function antagonistically for orchestrating the expression of key genes during development [65].

Transcription factors are also important regulators of gene expression. In *D. melanogaster*, transcription factors containing helix-loop-helix, C_2_H_2_ Zn-finger or forkhead DNA-binding domain play essential roles in developmental processes such as body plan determination and cell fate specification [66]. Interestingly, many genes coding for DNA-binding domain containing transcription factors were induced by TSA (Fig. 5c, Additional file 3: Excel File S2). A list of TSA response genes after various treatments is shown in Additional file 1: Figure S2. Interestingly, the expression levels of these genes decreased after *CBP* knockdown suggesting that acetylation levels maintained by HATs (*CBP*) and HDAC inhibitors (TSA) regulate the expression. Gene coding for Forkhead box protein O (*FoxO*), a transcription factor that plays important roles in the regulation of metabolism, development, and organogenesis through either activation or repression of the target gene expression, was induced by TSA (Additional file 3: Excel File S2). FoxO regulates lipolysis in fat body cells by regulating the expression of lipases (i.e., *brummer, acid lipase-1*) during molting and pupation in *Bombyx mori* [67]. Recently, Zeng et al., (2017) [68] documented the role of FoxO transcription factor in regulating JH degradation in *Bombyx mori*. However, the function of FoxO could vary among different insect orders. In *D. melanogaster* (Diptera), delayed metamorphosis was caused by overexpression of FoxO [69] whereas, RNAi of *FoxO* caused delayed pupation in *T. castaneum* (Coleoptera) [70]. FoxO also regulates the timing of pupation through regulating ecdysteroid biosynthesis in *T. castaneum*, [70]. Our previous studies also documented the regulation of *Vg* gene expression by FoxO and JH in *T. castaneum* [71].

Genes coding for nuclear receptors (NRs) including *Ftz transcription factor 1* (*Ftz-f1*), *Hormone receptor 51* (*HR51*), *Seven-up* (*SVP*) and *Hormone receptor 38* (*HR38*) were induced by TSA. The NRs play a fundamental role in embryonic development and metamorphosis [72–75]. For example, expression of NRs, such as *E75*, *HR3*, and *Ftz-f1* is crucial for the development of *D. melanogaster* embryos [76]. The NRs also serve as the early response genes in the 20E regulation of molting and metamorphosis in *D. melanogaster* [77]. During postembryonic development, the Ftz-f1 expression is required for successful molting and metamorphosis [78, 79]. Specifically, βFtz-f1 acts as a competence factor for stage-specific responses to 20E pulse that initiates the pupal molt [80]. Similarly, *D. melanogaster* HR38 regulates metamorphosis [81]. Although HR38 expression was not directly under the control of 20E, it participates in the 20E signaling pathway as an alternative partner of ultraspiracle (USP) [82]. Our previous RNAi-based studies demonstrated the role of *TcFtz-f1* and TcHR51 during larval-pupal metamorphosis. Precisely, 100% mortality before pupation was observed when *dsTcFtz-f1* and *dsTcHR51* were injected into one-day old final instar *T. castaneum* larvae [83]. Likewise, TcSVP and TcHR38 play crucial roles in both larval-pupal and pupal-adult metamorphosis as *TcSVP*, and *TcHR38* RNAi larvae died either during larval-pupal transition or pupal-adult transition [83]. A similar RNAi-based study from our lab further disclosed the importance of TcSVP in reproduction, as indicated by the observation that newly eclosed *T. castaneum* females injected with *dsTcSVP* caused a decrease in *TcVg* transcript levels and egg production [84]. Recently, the contribution of SVP and Ftz-f1 in JH biosynthesis was also documented in *B. germanica* [85].

Interestingly, JH signal transduction pathway genes including *SRC* and *Kr-h1* were also induced by TSA. However, *SRC* induction by TSA was not confirmed by RT-qPCR (Fig. 3). *Kr-h1*, a mediator of JH action during metamorphosis in most of the insects, was induced TSA. Remarkably, knockdown of *CBP* suppressed TSA induction of *Kr-h1* (Additional file 3: Excel File S2). The expression of *Met* gene was unchanged by TSA suggesting some degree of selectivity (Fig. 3). The present study also revealed that TSA induces *fatty acid synthase* (*FAS*) expression. The contribution of FAS to lipid accumulation during diapause preparation in insects has been reported. Recently, Tan et al., [86] found that *fatty acid synthase 2* contributed explicitly to lipid accumulation and stress tolerance gene expression in the beetle, *Colaphellus. bowringi*. Furthermore, the induction of *calcium channel flower* (LOC658279), *calcium-transporting ATPase type 2C member 1* (LOC658547), *calpain-C* (LOC662931), etc. after TSA application suggested the possibility of regulation of gene expression through altering cellular Ca^2+^ homeostasis. However, such possibilities need to be confirmed.

The widespread changes in gene expression after TSA treatment was also shown in the pathway enrichment analysis of 699 genes induced by TSA. Some metabolic pathways including MAPK, Hippo, FoxO, and Wnt signaling and fatty acid metabolism, Longevity regulating pathways and TGFβ signaling pathway were also enriched after TSA treatment (Additional file 1: Figure S3A). Among them, the TGF-β signaling pathway and FoxO signaling were shown to regulate insect metamorphosis [70, 87]. Thus TSA (alternatively HDACs) may indirectly involve in hormonal regulation during metamorphosis in *T. castaneum*.

### CBP regulates genes involved in the development and hormone action

**Fig. 6:**
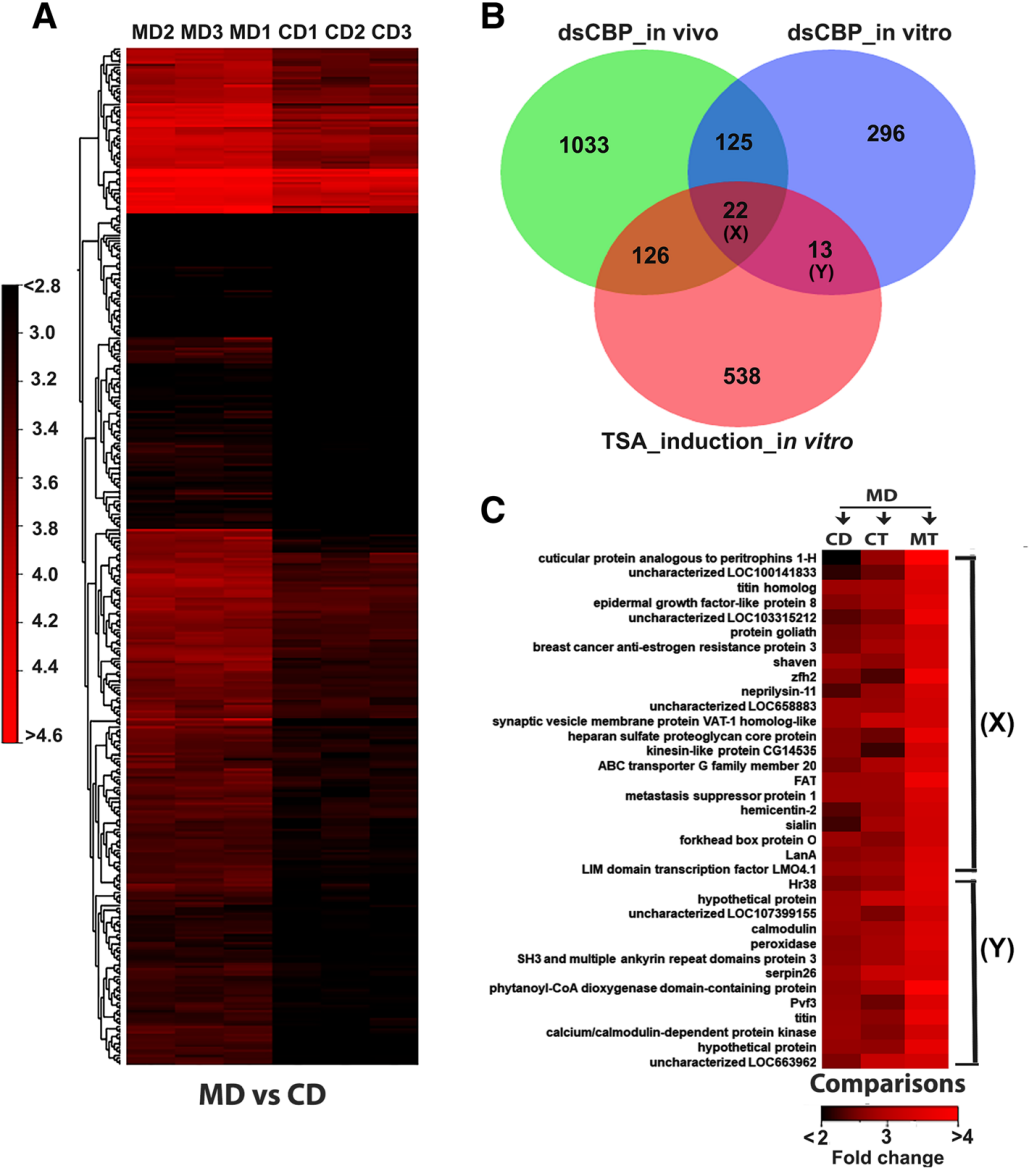
Impact of dsCBP on gene expression. (**a**) Heatmap illustrating the TMM normalized expression values of 456 down-regulated genes (*p* < 0.01, ≤ 2-fold ↓) after dsCBP treatment in TcA cells. (**b**) Venn diagram representing the genes that were commonly affected by dsCBP treatment in vitro, in vivo (data not shown), and induced after TSA treatment. Twenty-two genes (**X**) were common in between all the comparisons (MD vs. CD, MD vs. MT, dsCBP in vivo) whereas thirteen genes (**Y**) were common in between comparisons such as MD vs. CD and MD vs. MT. (**c**) Heatmap showing the expression of selected genes that were represented as ‘*X*’ and ‘*Y*’ in section (**b**) after treatments of interest. [MD: dsmalE treated cells exposed to DMSO; CD: dsCBP treated cells exposed to DMSO; MT: dsmalE treated cells exposed to TSA]

CBP is a transcriptional co-activator with HAT activity regulating the activity of hundreds of transcription factors. During postembryonic development, Nejire (a homolog of CBP), contributes to the ecdysone signaling pathway. Nejire regulates the expression of *sox14*, an ecdysone-response gene through acetylation of H3K27 [88]. Our previous in vivo studies in *T. castaneum* larvae indicated the role of CBP in insect postembryonic development [89]. However, due to interference from endogenous hormones, we did not obtain a satisfactory picture of the impact of *CBP* RNAi on epigenetic regulation of JH action. In the current study, we aimed to get a more comprehensive picture using JH and TSA treatments after *CBP* RNAi (Table 1). *CBP* knockdown (CD or *dsCBP* + DMSO) caused down-regulation of 456 genes in TcA cells compared to control cells exposed to *dsmalE* (MD or *dsmalE* + DMSO) (Fig. 6a, Additional file 4: Excel S3). Nearly one third (approximately 147) of the 456 genes were also found to be down-regulated in the *CBP* dsRNA injected *T. castaneum* larvae [89] (Fig. 6b, Additional file 7: Excel S6). Among these 147 genes, many of them such as *FoxO*, *fatty acid synthase*, *hairy* (*H*), *insulin receptor* (*InR*), *Laminin subunit alpha* (*LanA*), *adenosine deaminase*, *bambi*, *eyegone*, etc. are known to be involved in hormonal regulation of development and metamorphosis. The contributions of FoxO and fatty acid synthase in hormone biosynthesis (20E) in the regulation of metamorphosis, reproduction, and lipid biosynthesis during diapause preparation were already discussed in the previous section. Among the other genes, JH-response gene *hairy* (*H*) acts downstream to the Met in the JH action cascade in *Aedes aegypti* [90].

Insulin-like growth factor (IGF) signaling plays a vital role in insect growth, development and metabolism by controlling glucose uptake and protein translation. In *T. castaneum,* the two *InRs* play distinct roles during development and reproduction. Thus, down-regulation of *InR* by *CBP* knockdown could affect those processes, but such an assumption needs to be experimentally confirmed. *LanA* was another essential gene whose expression was affected by depletion of *CBP* both in larvae and in TcA cells. Loss of function mutations of lanA gene in *D. melanogaster* caused embryonic lethality, whereas individuals with holomorphic alleles in specific combination gave rise to escaper adults with reduced viability and tissue defects demonstrating the contribution of *LanA* during *D. melanogaster* development [91]. *Adenosine deaminase CECR1-A* (*Cat Eye Critical Region 1*) expression was also affected by *CBP* knockdown. In *D. melanogaster*, expression of adenosine deaminases increases during late larval stage suggesting that it may function during metamorphosis. The enzyme, adenosine deaminase may protect the tissues from toxic nucleosides released from cellular apoptosis during metamorphosis by transforming them into their respective inosine nucleosides and thus helping in tissue reconstruction during metamorphosis [92]. Reduced expression of these physiologically important genes by *CBP* RNAi could induce mortality during metamorphosis as reported recently [89].

Transcription factors such as *mad2* and *bambi* were also down-regulated in cells exposed to *dsCBP*. Bambi, a Bone Morphogenetic Protein (BMP) inhibitor present in *T. castaneum* and A*pis* genomes but not in *Drosophila*, was reported to be expressed in regions with high BMP signaling indicating its role in modulating TGFβ signaling [93]. In the dipteran model insect, *D. melanogaster*, TGF-β signaling induced JH production through up-regulation of *JH acid methyltransferase* (*JHAMT*) gene transcription through transcription factor Mothers Against Dpp (MAD) [94]. These data suggest that CBP could modulate the TGFβ signaling and JH biosynthesis by controlling the expression of bambi and JHAMT (via mad2). Similarly, the expression of Pax transcription factor gene *eyegone* (*eyg*) was also affected by depletion of *CBP*. In *T. castaneum*, *eyegone* is essential for normal development of hind wings, antennae, and eyes [95].

Some other key functional genes are affected in TcA cells after *CBP* RNAi treatment. For instance, a nuclear receptor, *HR38*, was down-regulated after *CBP* RNAi in these cells. Besides *H38*, the expression of other essential genes such as *hexokinase 2* (*HK2*), *Takeout*, *H17* and different cytochromes (i.e., *Cyt18A1*, *Cyt303A1*, *Cyp4q6*, *Cyp4C1*, etc.) that are known to be involved in various physiological function in insects, were affected by *CBP* RNAi. HK2 was one of the key components in insect chitin biosynthesis pathway that contributes to insect growth and development [96, 97]. In *T. castaneum,* RNAi of *trehalose- 6-phosphate synthase* (TcTPS) significantly reduced the expression of HK2, and the larvae could not complete the transition from larvae to pupae due to a lower level of chitin and resulted in abnormal phenotypes [98]. Hence, down-regulation of *HK2* could also lead to abnormal molting phenotype by negatively influencing chitin synthesis in *T. castaneum* as observed in our recent study where *CBP* was knocked down in *T. castaneum* larvae [89]. Similarly, the expression of *takeout* gene, reported to be regulated by JH [99], was also affected by *CBP* RNAi. Interestingly, in *N. lugens*, the expression of the *takeout* gene increased after knockdown of *Met* and *Kr-h1* genes and decreased by RNAi of *Met* interacting partners such as *NITai* and *NIβ-Ftz-f1* [99]. Although the depletion of *CBP* has no direct effect on *Met* expression pattern, the indirect involvement of CBP via takeout in JH signaling pathway mediated by Met and other Met interacting partners is possible but needs to be experimentally validated.

Two genes, *H17* and *farnesol dehydrogenase*, which play antagonistic roles in JH biosynthesis, were also affected by *CBP* RNAi. H17, a mimic of the allatostatin A, inhibits JH biosynthesis in *B. germanica* and thus acts as an insect growth regulator (IGR) [100]. The influence of allatostatin A on vitellogenesis was also documented in *B. germanica* [101]. *CBP* RNAi also affected *farnesol dehydrogenase* expression. Oxidation of NADP-dependent farnesol dehydrogenase is a rate-limiting step in JH biosynthesis pathway in adult mosquitoes [102]. The decrease in farnesol dehydrogenase expression could lead to reduced endogenous JH synthesis. Hence, CBP may also influence JH production in insects. Another gene family, *Cytochrome P450s* (*CYP*s), perform vital physiological functions during all life stages of an insect through their involvement in the biosynthesis of 20E and JH [103, 104]. The expression of different *cytochrome P450* family members was downregulated by *CBP* RNAi. For example, the expression of *CYP18A1* that is essential for *D. melanogaster* growth and development was reduced after *CBP* RNAi [105]. Some of the genes from a *CYP4* family that is known to be involved in the metabolism of JH III were also downregulated by *CBP* knockdown [106]. Furthermore, RNAi of *CBP* decreased the expression of transcripts such as *calcium-activated chloride channel regulator 4* (LOC662351), *sodium/potassium-transporting ATPase subunit alpha* (LOC663833) and *calcium release-activated calcium channel protein* 1(LOC657890) possibly suggesting a role in the maintenance of cellular Ca^2+^ homeostasis. However, additional experiments are needed to test this hypothesis.

Finally, Pathway enrichment analysis of the 456 downregulated genes revealed the signaling pathways regulated by CBP (Additional file 1: Figure S3 B). There were many pathways, related to insect growth, immunity and development, affected by *CBP* RNAi. For instance, FoxO signaling, fatty acid metabolism, Insect hormone biosynthesis, Toll and IMD pathway, TGF-β, and Hedgehog signaling pathways were among the pathways enriched > 1.5 times in *CBP* RNAi samples. Functionally, FoxO signaling, insect hormone biosynthesis, and TGF-β signaling are involved in insect hormone biosynthesis; reproduction and metamorphosis, Toll and IMD pathway are involved in insect immunity development and hedgehog signaling pathway involved in adult appendages patterning [87, 107–111].

### Functional correlation between CBP RNAi and TSA treatment

Both CBP and TSA work as epigenetic modulators of gene expression through their different roles as writer and eraser of acetylation. The expression of some genes was regulated by both CBP and TSA. We identified 35 genes that were regulated by both CBP and TSA (Fig. 6b, Additional file 7: Excel S6). The application of TSA after *CBP* knockdown could not rescue the expression of these genes. Importantly, *FoxO* and *HR38* that are known to mediate JH and 20E signaling respectively were affected by both CBP and TSA. These data suggest that acetylation (by HATs, e.g., CBP) and deacetylation (HDACs, TSA studies) could regulate expression of key players involved in JH and 20E action thus adding an extra layer of epigenetic regulation to hormone action (Fig. 6c, Additional file 7: Excel File S6). Pathway enrichment analysis has also revealed the regulation of common pathways by both CBP and TSA. Pathways such as TGF-β, FoxO, and Wnt signaling, ECM receptor interaction and fatty acid biosynthesis were regulated by both CBP and TSA in TcA cells. Interestingly, comparing the CBP RNAi in *T. castaneum* in larvae [89] and TSA treatment of TcA cells, we found 148 genes that were affected by both these treatments. Among them, *Kr-h1*, *svp*, *Fz2*, *polycomb*, *lanA*, FoxO and *BMP receptor type-1B* were shown to regulate insect development and metamorphosis (Additional file 7: Excel S6). Moreover, ~ 22 genes including *FoxO* and *lanA* responded to *CBP* RNAi in larvae and TcA cells as well as TSA treatment in TcA cells (Fig. 6b and c; Additional file 7: excel S6). The expression levels of *FoxO* was further validated by RT-qPCR (Additional file 1: Figure S4). The function of *FoxO* in *T. castaneum* growth, reproduction and metamorphosis has been reported previously, but the report on epigenetic regulation of *FoxO* through CBP and HDACs is one of the major contributions of this paper. However, the effect of epigenetic regulation of *FoxO* on hormone action requires further studies.

## Conclusions

JH and 20E play vital roles in insect life by orchestrating the molting and metamorphosis processes. Synthesis of these hormones and their spatiotemporal action are indispensable for successful molting and metamorphosis. Such level of precision in the synthesis of these hormones and expression of their downstream response genes during different life stages of insects indeed demands a higher degree of transcriptional as well as translational regulation. The present study shows the function of CBP and HDAC in regulating JH hormone action and corroborates our previous findings on *T. castaneum* larvae with only *CBP* RNAi treatment. The impact of *dsCBP* and TSA on the expression of the JH response gene, *Kr-h1*, was confirmed. The expression of *Vg* gene was also affected by *dsCBP* to such an extent that the expression level did not recover by further JH treatment. The expression of other key developmental genes such as *bambi*, *eyegone*, *FoxO*, *lanA*, *svp*, *polycomb*, *Fz-2*, *HR38* and *hairy* was also influenced by the *CBP* RNAi and/or HDAC inhibition. Many key physiological pathways involved in insect growth and development such as TGF-β and FoxO signaling were modulated by both treatments. Thus, CBP and HDAC may contribute significantly to the hormonal regulation of *T. castaneum* metamorphosis. Interestingly, *FoxO* gene expression was significantly altered after *CBP* RNAi or TSA treatment confirming the epigenetic regulation of this important transcription factor. Further studies based on chip sequencing and functional proteomics will help to elucidate the precise mechanisms involved in epigenetic regulation of JH action in insects.

## Additional files

Additional file 1: **Table S1.** List of primers. **Table S2.** Detailed RNA seq statistics. Table is showing a summary of read statistics after Illumina Hi-seq 4000 sequencing for individual biological replicates in different treatments. **Figure S1.** Correlation of gene expression levels of selected genes (15) by comparing both RT-qPCR and RNA-seq data. Individual log fold changes obtained by RT-qPCR and RNA-seq for each gene in the sample group. **Figure S2.** Relative expression of the epi-factor domain-containing genes in MD vs. MT, MD vs. CD and MD vs. CT comparisons. Heatmaps are illustrating the relative expression of genes with the epi-factor domain, in all three treatments (MT, CD, and CT). **Figure S3.** Pathway enrichment analysis. A) Pathways enriched after TSA induction. B) Pathway affected due to CBP RNAi. Here, the negative impact is equivalent to the enrichment. **Figure S4.** Relative expression of FoxO transcription factor after different treatments in TcA cells and CBP RNAi in larvae. The data shown are the mean + S.E. (*n* = 4). (Letters represent significance at 95% CI). (PDF 9301 kb)

Additional file 2: **Excel S1.** Excel sheet-containing details of 32 genes induced by JH III (MD vs. MJ) and their relative expression in all other treatments compared to control. Genes behaving like Kr-h1 showed separately in a second excel sheet. (XLSX 23 kb)

Additional file 3: **Excel S2.** Excel sheet containing details of 699 genes induced by TSA (MD vs. MT) and their relative expression in other treatments of interest such as MD vs. CD and MD vs. CT. Genes with epifactor domains showed separately in a second excel sheet. Expression of Met and SRC in all three treatments showed in another excel sheet. (XLSX 133 kb)

Additional file 4: **Excel S3.** Excel sheet containing details of 456 genes suppressed by CBP RNAi (MD vs. CD) and their relative expression in all other comparisons such as MD vs. CT, MD vs. MJ, MD vs. CJ and MD vs. MT. (XLSX 89 kb)

Additional file 5: **Excel S4.** Excel sheet containing details of 181 genes responded after CBP RNAi and subsequent JH III treatment. (XLS 69 kb)

Additional file 6: **Excel S5.** Excel sheet containing details of 602 genes responded after CBP RNAi and subsequent TSA treatment. (XLS 132 kb)

Additional file 7: **Excel S6.** Excel sheet containing details of gene clusters obtained after Venn diagram analysis shown in main Fig. 6b. (XLSX 43 kb)

## Abbreviations

20E: 20-hydroxyecdysone
CD: dsCBP treated cells exposed to DMSO
CJ: dsCBP treated cells exposed to JH III
CT: dsCBP treated cells exposed to TSA
dsCBP: dsRNA targeting a fragment of CREB-binding protein
dsmalE: dsRNA targeting a fragment of *E. coli* maltase gene
EcR: Ecdysone receptor
FOXO: Forkhead transcription factor
GPCR: G-protein-coupled receptor
HATs: Histone acetyltransferases
HDACs: Histone deacetylases
JHAMT: JH acid methyltransferase
JHIII: Juvenile Hormone III
Kr-h1: Krüppel homolog 1
MD: dsmalE treated cells exposed to DMSO
Met: Methoprene-tolerant
MJ: dsmalE treated cells exposed to JH III
Mmp-3: Matrix metalloproteinase-3
MT: dsmalE treated cells exposed to TSA
nAChRa9: Nicotinic acetylcholine receptor alpha9 subunit
SRC: Steroid receptor coactivator
TcA cells: *Tribolium castaneum* cells
TSA: Trichostatin A

## References

[1] Buszczak M, Segraves WA (2000). Insect metamorphosis: out with the old, in with the new. Curr Biol.

[2] Truman JW, Riddiford LM (2002). Endocrine insights into the evolution of metamorphosis in insects. Annu Rev Entomol.

[3] Yamanaka N, Rewitz KF, O'Connor MB (2013). Ecdysone control of developmental transitions: lessons from Drosophila research. Annu Rev Entomol.

[4] Dubrovsky EB (2005). Hormonal cross talk in insect development. Trends Endocrinol Metab.

[5] Nakagawa Y, Henrich VC (2009). Arthropod nuclear receptors and their role in molting. FEBS J.

[6] Riddiford LM (2012). How does juvenile hormone control insect metamorphosis and reproduction?. Gen Comp Endocrinol.

[7] Jindra M, Palli SR, Riddiford LM (2013). The juvenile hormone signaling pathway in insect development. Annu Rev Entomol.

[8] Kayukawa T, Jouraku A, Ito Y, Shinoda T. Molecular mechanism underlying juvenile hormone-mediated repression of precocious larval–adult metamorphosis. Proc Natl Acad Sci U S A. 2017;201615423. 10.1073/pnas.1615423114 PMC5293048 28096379

[9] Liu P, Peng H-J, Zhu J (2015). Juvenile hormone-activated phospholipase C pathway enhances transcriptional activation by the methoprene-tolerant protein. Proc Natl Acad Sci U S A.

[10] Li K, Jia QQ, Li S. Juvenile hormone signaling–a mini review. Insect science. 2018. 10.1111/1744-7917.12614 29888456

[11] Bai H, Palli SR (2016). Identification of G protein-coupled receptors required for vitellogenin uptake into the oocytes of the red flour beetle, *Tribolium castaneum*. Sci Rep.

[12] Cai M-J, Dong D-J, Wang Y, Liu P-C, Liu W, Wang J-X, Zhao X-F (2014). G-protein-coupled receptor participates in 20-hydroxyecdysone signaling on the plasma membrane. Cell Communication and Signaling.

[13] Luft UC, Bychkov R, Gollasch M, Gross V, Roullet J-B, McCarron DA, Ried C, Hofmann F, Yagil Y, Yagil C (1999). Farnesol blocks the L-type Ca2+ channel by targeting the α1C subunit. Arterioscler Thromb Vasc Biol.

[14] Wyatt GR, Davey KG (1996). Cellular and molecular actions of juvenile hormone. II. Roles of juvenile hormone in adult insects. Adv Insect Physiol.

[15] Soller M, Bownes M, Kubli E (1999). Control of oocyte maturation in sexually matureDrosophilafemales. Dev Biol.

[16] Herndon L, Chapman T, Kalb J, Lewin S, Partridge L, Wolfner M (1997). Mating and hormonal triggers regulate accessory gland gene expression in male Drosophila. J Insect Physiol.

[17] Belgacem YH, Martin J-R (2002). Neuroendocrine control of a sexually dimorphic behavior by a few neurons of the pars intercerebralis in Drosophila. Proc Natl Acad Sci U S A.

[18] Parthasarathy R, Tan A, Sun Z, Chen Z, Rankin M, Palli SR (2009). Juvenile hormone regulation of male accessory gland activity in the red flour beetle, *Tribolium castaneum*. Mech Dev.

[19] Akimaru H, Chen Y, Dai P, Hou D-X, Nonaka M, Smolik SM, Armstrong S, Goodman RH, Ishii S (1997). *Drosophila* CBP is a co-activator of cubitus interruptus in hedgehog signalling. Nature.

[20] Akimaru H, Hou D-X, Ishii S (1997). Drosophila CBP is required for dorsal–dependent twist gene expression. Nat Genet.

[21] Hung H-C, Maurer C, Kay SA, Weber F (2007). Circadian transcription depends on limiting amounts of the transcription co-activator nejire/CBP. J Biol Chem.

[22] McManus KJ, Hendzel MJ (2001). CBP, a transcriptional coactivator and acetyltransferase. Biochem Cell Biol.

[23] Goodman RH, Smolik S (2000). CBP/p300 in cell growth, transformation, and development. Genes Dev.

[24] Ylla G, Belles X (2015). Corrigendum to “towards understanding the molecular basis of cockroach tergal gland morphogenesis. A transcriptomic approach”[insect Biochem. Mol. Biol. 63 (2015) 104–112]. Insect Biochem Mol Biol.

[25] Spannhoff A, Kim YK, Raynal NJM, Gharibyan V, Su MB, Zhou YY, Li J, Castellano S, Sbardella G, Issa JPJ (2011). Histone deacetylase inhibitor activity in royal jelly might facilitate caste switching in bees. EMBO Rep.

[26] Lockett GA, Wilkes F, Helliwell P, Maleszka R (2014). Contrasting effects of histone deacetylase inhibitors on reward and aversive olfactory memories in the honey bee. Insects.

[27] Ozawa T, Mizuhara T, Arata M, Shimada M, Niimi T, Okada K, Okada Y, Ohta K. Histone deacetylases control module-specific phenotypic plasticity in beetle weapons. Proc Natl Acad Sci. 2016;113(52):15042-7. 10.1073/pnas.1615688114 PMC5206575 27956627

[28] Simola DF, Graham RJ, Brady CM, Enzmann BL, Desplan C, Ray A, Zwiebel LJ, Bonasio R, Reinberg D, Liebig J (2016). Epigenetic (re) programming of caste-specific behavior in the ant *Camponotus floridanus*. Science.

[29] Kayukawa T, Tateishi K, Shinoda T (2013). Establishment of a versatile cell line for juvenile hormone signaling analysis in *Tribolium castaneum*. Sci Rep.

[30] Xu J, Roy A, Palli SR (2018). CREB-binding protein plays key roles in juvenile hormone action in the red flour beetle, *Tribolium Castaneum*. Sci Rep.

[31] Goodman CL, Stanley D, Ringbauer JA, RW JB, Silver K, Park Y (2012). A cell line derived from the red flour beetle *Tribolium castaneum* (Coleoptera: Tenebrionidae). In vitro cellular & developmental biology Animal.

[32] Zhang Z, Xu J, Sheng Z, Sui Y, Palli SR (2011). Steroid receptor co-activator is required for juvenile hormone signal transduction through a bHLH-PAS transcription factor, methoprene tolerant. J Biol Chem.

[33] Kalsi M, Palli SR (2017). Cap n collar transcription factor regulates multiple genes coding for proteins involved in insecticide detoxification in the red flour beetle, *Tribolium castaneum*. Insect Biochem Mol Biol.

[34] Ma L, Pati PK, Liu M, Li QQ, Hunt AG (2014). High throughput characterizations of poly (a) site choice in plants. Methods.

[35] Richards S, Gibbs RA, Weinstock GM, Brown SJ, Denell R, Beeman RW, Gibbs R, Bucher G, Friedrich M, Grimmelikhuijzen CJ (2008). The genome of the model beetle and pest *Tribolium castaneum*. Nature.

[36] Roy A, Walker WB, Vogel H, Kushwaha S, Chattington S, Larsson M, Anderson P, Heckel D, Schlyter F (2016). Data set for diet specific differential gene expression analysis in three Spodoptera moths. Data in brief.

[37] Kalsi M, Palli SR (2015). Transcription factors, CncC and Maf, regulate expression of CYP6BQ genes responsible for deltamethrin resistance in *Tribolium castaneum*. Insect Biochem Mol Biol.

[38] Parthasarathy R, Tan A, Palli SR. bHLH-PAS family transcription factor methoprene-tolerant plays a key role in JH action in preventing the premature development of adult structures during larval-pupal metamorphosis. Mech Dev. 2008;125:601-16. 10.1016/j.mod.2008.03.004 PMC2486318 18450431

[39] Xu J, Sheng Z, Palli SR (2013). Juvenile hormone and insulin regulate trehalose homeostasis in the red flour beetle, *Tribolium castaneum*. PLoS Genet.

[40] Nijhout HF. Insect Hormones. Princeton: Princeton Univ. Press; 1994.

[41] Staal G (1986). Anti juvenile hormone agents. Annu Rev Entomol.

[42] Tan A, Tanaka H, Tamura T, Shiotsuki T (2005). Precocious metamorphosis in transgenic silkworms overexpressing juvenile hormone esterase. Proc Natl Acad Sci U S A.

[43] Minakuchi C, Namiki T, Yoshiyama M, Shinoda T (2008). RNAi-mediated knockdown of juvenile hormone acid O-methyltransferase gene causes precocious metamorphosis in the red flour beetle *Tribolium castaneum*. FEBS J.

[44] Kayukawa T, Murata M, Kobayashi I, Muramatsu D, Okada C, Uchino K, Sezutsu H, Kiuchi M, Tamura T, Hiruma K (2014). Hormonal regulation and developmental role of Krüppel homolog 1, a repressor of metamorphosis, in the silkworm Bombyx mori. Dev Biol.

[45] Minakuchi C, Namiki T, Shinoda T (2009). Kruppel homolog 1, an early juvenile hormone-response gene downstream of Methoprene-tolerant, mediates its anti-metamorphic action in the red flour beetle *Tribolium castaneum*. Dev Biol.

[46] Minakuchi C, Zhou X, Riddiford LM (2008). Krüppel homolog 1 (Kr-h1) mediates juvenile hormone action during metamorphosis of Drosophila melanogaster. Mech Dev.

[47] Parthasarathy R, Tan A, Bai H, Palli SR (2008). Transcription factor broad suppresses precocious development of adult structures during larval-pupal metamorphosis in the red flour beetle, *Tribolium castaneum*. Mech Dev.

[48] Zhou B, Hiruma K, Shinoda T, Riddiford LM (1998). Juvenile hormone prevents ecdysteroid-induced expression of broad complex RNAs in the epidermis of the tobacco hornworm, *Manduca sexta*. Dev Biol.

[49] Dushay MS, Asling B, Hultmark D (1996). Origins of immunity: relish, a compound Rel-like gene in the antibacterial defense of Drosophila. Proc Natl Acad Sci U S A.

[50] Mosavi LK, Cammett TJ, Desrosiers DC, Zy P (2004). The ankyrin repeat as molecular architecture for protein recognition. Protein Sci.

[51] Voronin D, Kiseleva E (2008). Functional role of proteins containing ankyrin repeats. Cell Tiss Biol.

[52] Flatt T, Heyland A, Rus F, Porpiglia E, Sherlock C, Yamamoto R, Garbuzov A, Palli SR, Tatar M, Silverman N (2008). Hormonal regulation of the humoral innate immune response in *Drosophila melanogaster*. J Exp Biol.

[53] Sheng Z, Xu J, Bai H, Zhu F, Palli SR (2011). Juvenile hormone regulates vitellogenin gene expression through insulin-like peptide signaling pathway in the red flour beetle, *Tribolium castaneum*. J Biol Chem.

[54] Comas D, Piulachs M-D, Bellés X (2001). Induction of vitellogenin gene transcription in vitro by juvenile hormone in Blattella germanica. Mol Cell Endocrinol.

[55] Chinzei Y, White B, Wyatt G (1982). Vitellogenin mRNA in locust fat body: identification, isolation, and quantitative changes induced by juvenile hormone. Can J Biochem.

[56] Llano E, Adam G, Pendás AM, Quesada V, Sánchez LM, Santamaría I, Noselli S, López-Otín C (2002). Structural and enzymatic characterization of Drosophila Dm2-MMP, a membrane-bound matrix metalloproteinase with tissue-specific expression. J Biol Chem.

[57] Llano E, Pendás AM, Aza-Blanc P, Kornberg TB, López-Otín C (2000). Dm1-MMP, a matrix metalloproteinase fromDrosophila with a potential role in extracellular matrix remodeling during neural development. J Biol Chem.

[58] Knorr E, Schmidtberg H, Vilcinskas A, Altincicek B (2009). MMPs regulate both development and immunity in the *Tribolium* model insect. PLoS One.

[59] Sevala VL, Davey K, Prestwich GD (1995). Photoaffinity labeling and characterization of a juvenile hormone binding protein in the membranes of follicle cells of Locusta migratoria. Insect Biochem Mol Biol.

[60] Xiao W, Chen X, Liu X, Luo L, Ye S, Liu Y (2014). Trichostatin a, a histone deacetylase inhibitor, suppresses proliferation and epithelial–mesenchymal transition in retinal pigment epithelium cells. J Cellular Mol Med.

[61] Medvedeva YA, Lennartsson A, Ehsani R, Kulakovskiy IV, Vorontsov IE, Panahandeh P, Khimulya G, Kasukawa T, Consortium F, Drabløs F (2015). EpiFactors: a comprehensive database of human epigenetic factors and complexes. Database.

[62] Dillon SC, Zhang X, Trievel RC, Cheng X (2005). The SET-domain protein superfamily: protein lysine methyltransferases. Genome Biol.

[63] Takeuchi T, Watanabe Y, Takano-Shimizu T, Kondo S (2006). Roles of jumonji and jumonji family genes in chromatin regulation and development. Dev Dyn.

[64] Vidal NM, Grazziotin AL, Iyer LM, Aravind L, Venancio TM (2016). Transcription factors, chromatin proteins and the diversification of Hemiptera. Insect Biochem Mol Biol.

[65] Schuettengruber B, Chourrout D, Vervoort M, Leblanc B, Cavalli G (2007). Genome regulation by polycomb and trithorax proteins. Cell.

[66] McGinnis W, Krumlauf R (1992). Homeobox genes and axial patterning. Cell.

[67] Hossain MS, Liu Y, Zhou S, Li K, Tian L, Li S (2013). 20-Hydroxyecdysone-induced transcriptional activity of FoxO upregulates brummer and acid lipase-1 and promotes lipolysis in Bombyx fat body. Insect Biochem Mol Biol.

[68] Zeng B, Huang Y, Xu J, Shiotsuki T, Bai H, Palli SR, Huang Y, Tan A. The FOXO transcription factor controls insect growth and development by regulating juvenile hormone degradation in the silkworm, Bombyx mori. J Biol Chem. 2017. 10.1074/jbc.M117.777797. 10.1074/jbc.M117.777797 PMC5512063 28490635

[69] Koyama T, Rodrigues MA, Athanasiadis A, Shingleton AW, Mirth CK (2014). Nutritional control of body size through FoxO-Ultraspiracle mediated ecdysone biosynthesis. Elife.

[70] Lin X, Yu N, Smagghe G (2017). FoxO mediates the timing of pupation through regulating ecdysteroid biosynthesis in the red flour beetle, *Tribolium castaneum*. Gen comp Endocrinol.

[71] Parthasarathy R, Palli SR (2011). Molecular analysis of nutritional and hormonal regulation of female reproduction in the red flour beetle, *Tribolium castaneum*. Insect Biochem Mol Biol.

[72] DeLuca HF (2004). Overview of general physiologic features and functions of vitamin D. American J Clin Nutr.

[73] Martín D. Nuclear Receptor. In: Functions of nuclear receptors in insect development. Dordrecht, Heidelberg, London, New York: Springer; 2010. p. 31–61.

[74] Fahrbach SE, Smagghe G, Velarde RA (2012). Insect nuclear receptors. Annu Rev Entomol.

[75] Jarvela AMC, Pick L (2017). Chapter two-the function and evolution of nuclear receptors in insect embryonic development. Curr Top Dev Biol.

[76] Carney GE, Wade AA, Sapra R, Goldstein ES, Bender M (1997). DHR3, an ecdysone-inducible early-late gene encoding a *Drosophila* nuclear receptor, is required for embryogenesis. Proc Natl Acad Sci U S A.

[77] Sullivan AA, Thummel CS (2003). Temporal profiles of nuclear receptor gene expression reveal coordinate transcriptional responses during *Drosophila* development. Mol Endocrinol.

[78] M-a Y, Murata T, Hirose S, Lavorgna G, Suzuki E, Ueda H (2000). Temporally restricted expression of transcription factor betaFTZ-F1: significance for embryogenesis, molting and metamorphosis in *Drosophila melanogaster*. Development.

[79] Cruz J, Martín D, Bellés X (2007). Redundant ecdysis regulatory functions of three nuclear receptor HR3 isoforms in the direct-developing insect *Blattella germanica*. Mech Dev.

[80] Broadus J, McCabe JR, Endrizzi B, Thummel CS, Woodard CT (1999). The *Drosophila* βFTZ-F1 orphan nuclear receptor provides competence for stage-specific responses to the steroid hormone ecdysone. Mol Cell.

[81] Kozlova T, Pokholkova G, Tzertzinis G, Sutherland J, Zhimulev I, Kafatos F (1998). *Drosophila* hormone receptor 38 functions in metamorphosis: a role in adult cuticle formation. Genetics.

[82] Baker KD, Shewchuk LM, Kozlova T, Makishima M, Hassell A, Wisely B, Caravella JA, Lambert MH, Reinking JL, Krause H (2003). The *Drosophila* orphan nuclear receptor DHR38 mediates an atypical ecdysteroid signaling pathway. Cell.

[83] Tan A, Palli SR (2008). Identification and characterization of nuclear receptors from the red flour beetle, *Tribolium castaneum*. Insect Biochem Mol Biol.

[84] Xu J, Tan A, Palli SR (2010). The function of nuclear receptors in regulation of female reproduction and embryogenesis in the red flour beetle, *Tribolium castaneum*. J Insect Physiol.

[85] Borras-Castells F, Nieva C, Maestro JL, Maestro O, Belles X, Martín D (2017). Juvenile hormone biosynthesis in adult *Blattella germanica* requires nuclear receptors seven-up and FTZ-F1. Sci Rep.

[86] Tan Q-Q, Liu W, Zhu F, Lei C-L, Wang X-P (2017). Fatty acid synthase 2 contributes to diapause preparation in a beetle by regulating lipid accumulation and stress tolerance genes expression. Sci Rep.

[87] Ishimaru Y, Tomonari S, Matsuoka Y, Watanabe T, Miyawaki K, Bando T, Tomioka K, Ohuchi H, Noji S, Mito T (2016). TGF-β signaling in insects regulates metamorphosis via juvenile hormone biosynthesis. Proc Natl Acad Sci U S A.

[88] Kirilly D, Wong JJ, Lim EK, Wang Y, Zhang H, Wang C, Liao Q, Wang H, Liou YC, Yu F (2011). Intrinsic epigenetic factors cooperate with the steroid hormone ecdysone to govern dendrite pruning in *Drosophila*. Neuron.

[89] Roy A, George S, Palli SR (2017). Multiple functions of CREB-binding protein during postembryonic development: identification of target genes. BMC Genomics.

[90] Saha TT, Shin SW, Dou W, Roy S, Zhao B, Hou Y, Wang XL, Zou Z, Girke T, Raikhel AS (2016). Hairy and Groucho mediate the action of juvenile hormone receptor Methoprene-tolerant in gene repression. Proc Natl Acad Sci U S A.

[91] Henchcliffe C, García-Alonso L, Tang J, Goodman CS (1993). Genetic analysis of laminin a reveals diverse functions during morphogenesis in Drosophila. Development.

[92] Dolezelova E, Zurovec M, Dolezal T, Simek P, Bryant PJ (2005). The emerging role of adenosine deaminases in insects. Insect Biochem Mol Biol.

[93] Van der Zee M, Da Fonseca RN, Roth S (2008). TGFβ signaling in Tribolium: vertebrate-like components in a beetle. Dev Genes Evol.

[94] Huang J, Tian L, Peng C, Abdou M, Wen D, Wang Y, Li S, Wang J (2011). DPP-mediated TGFβ signaling regulates juvenile hormone biosynthesis by activating the expression of juvenile hormone acid methyltransferase. Development.

[95] Zarin KN, Yang X, Bao R, Friedrich F, Beutel R, Friedrich M (2011). The Pax gene eyegone facilitates repression of eye development in Tribolium. EvoDevo.

[96] Tang B, Chen J, Yao Q, Pan Z, Xu W, Wang S, Zhang W (2010). Characterization of a trehalose-6-phosphate synthase gene from Spodoptera exigua and its function identification through RNA interference. J Insect Physiol.

[97] Chen J, Tang B, Chen H, Yao Q, Huang X, Chen J, Zhang D, Zhang W (2010). Different functions of the insect soluble and membrane-bound trehalase genes in chitin biosynthesis revealed by RNA interference. PLoS One.

[98] Chen Q, Jin S, Zhang L, Shen Q, Wei P, Wei Z, Wang S, Tang B. Regulatory functions of trehalose-6-phosphate synthase in the chitin biosynthesis pathway in *Tribolium castaneum* (Coleoptera: Tenebrionidae) revealed by RNA interference. Bulletin of Entomol Res. 2017:1–12. 10.1017/S000748531700089X 28920565

[99] Lin X, Zhang L, Jiang Y. Distinct roles of met and interacting proteins on the expressions of takeout family genes in Brown Planthopper. Front Physiol. 2017;8. 10.3389/fphys.2017.00100 PMC5318429 28270774

[100] Z-p K, Huang J, Tobe SS, X-l Y (2009). A potential insect growth regulator: synthesis and bioactivity of an allatostatin mimic. Peptides.

[101] Martin D, Piulachs M, Bellés X (1996). Inhibition of vitellogenin production by allatostatin in the German cockroach. Mol Cell Endocrinol.

[102] Mayoral JG, Nouzova M, Navare A, Noriega FG (2009). NADP+−dependent farnesol dehydrogenase, a corpora allata enzyme involved in juvenile hormone synthesis. Proc Natl Acad Sci U S A.

[103] Rewitz K, Yamanaka N, O'Connor MB (2013). Ecdysone control of developmental transitions: lessons from Drosophila research. Annu Rev Entomol.

[104] Helvig C, Koener J, Unnithan G, Feyereisen R (2004). CYP15A1, the cytochrome P450 that catalyzes epoxidation of methyl farnesoate to juvenile hormone III in cockroach corpora allata. Proc Natl Acad Sci U S A.

[105] Guittard E, Blais C, Maria A, Parvy J-P, Pasricha S, Lumb C, Lafont R, Daborn PJ, Dauphin-Villemant C (2011). CYP18A1, a key enzyme of Drosophila steroid hormone inactivation, is essential for metamorphosis. Dev Biol.

[106] Sutherland T, Unnithan G, Andersen J, Evans P, Murataliev M, Szabo L, Mash E, Bowers W, Feyereisen R (1998). A cytochrome P450 terpenoid hydroxylase linked to the suppression of insect juvenile hormone synthesis. Proc Natl Acad Sci U S A.

[107] Darakananda K. The role of the hedgehog signaling pathway in the regulation of larval and adult appendage patterning in the flour beetle, *Tribolium Castaneum*. Honors Thesis Collection. 2014:205.

[108] Süren-Castillo S, Abrisqueta M, Maestro JL (2012). FoxO inhibits juvenile hormone biosynthesis and vitellogenin production in the German cockroach. Insect Biochem Mol Biol.

[109] Bellés X, Martín D, Piulachs M-D (2005). The mevalonate pathway and the synthesis of juvenile hormone in insects. Annu Rev Entomol.

[110] Tanji T, Ip YT (2005). Regulators of the toll and Imd pathways in the *Drosophila* innate immune response. Trends Immunol.

[111] Noriega FG (2014). Juvenile hormone biosynthesis in insects: what is new, what do we know, and what questions remain?. Int Sch Res Notices.

