# Supplementary material for: Epigenetic modifications acetylation and deacetylation play important roles in juvenile hormone action
Source: BMC Genomics. 2018 Dec 14;19:934. doi: 10.1186/s12864-018-5323-4 (PMC6295036; doi:10.1186/s12864-018-5323-4)

Supplementary Information

Title: **Epigenetic modification of histones play important role in juvenile hormone action**

Amit Roy [1, 2] and Subba Reddy Palli[1]*

*Corresponding author: rpalli@uky.edu

[1]Department of Entomology, College of Agriculture, University of Kentucky, Lexington, KY 40546, USA

[2]Faculty of Forestry and Wood Sciences, EXTEMIT-K, Czech University of Life Sciences, Kamýcká 1176, Prague 6, Suchdol 165 21, Czech Republic

Supplementary Figure legends

Table S1: List of primers.

Table S2: Detailed RNA seq statistics. Table showing a summary of read statistics after Illumina Hi-seq 4000 sequencing for individual biological replicates in different treatments.

Figure S1: Correlation of gene expression levels of selected genes (15) by comparing both RT-qPCR and RNA-seq data. Individual log fold changes obtained by RT-qPCR and RNA-seq for each gene in the sample group.

Figure S2: Relative expression of the epi-factor domain-containing genes in MD (*dsmalE* + DMSO)vs. MT (*dsmalE* + TSA), MD (*dsmalE* + DMSO) vs. CD (*dsCBP* + DMSO) and MD (*dsmalE* + DMSO) vs. CT (*dsCBP* + TSA) comparisons**.** Heatmaps illustrating the relative expression of genes with the epi-factor domain, in all three treatments (MT, CD, CT).

Figure S3: Pathway enrichment analysis. A) Pathways enriched after TSA induction. B) Pathway affected due to CBP RNAi. Here, the negative impact is equivalent to the enrichment.

Figure S4: Relative expression of FoxO transcription factor after different treatments in TcA cells and CBP RNAi in larvae. The data shown are the mean + S.E. (n=4). (Letters represent significance at 95% CI).

Supplementary Excel S1: Excel sheet-containing details of 32 genes induced by JH III (MD or *dsmalE* + DMSO vs. MJ or *dsmalE* + JHIII) and their relative expression in all other treatments compared to control. Genes behaving like Kr-h1 showed separately in a second excel sheet.

Supplementary Excel S2: Excel sheet containing details of 699 genes induced by TSA (MD or *dsmalE* + DMSO vs. MT or *dsmalE* + TSA) and their relative expression in other treatments of interest such as MD vs. CD (*dsCBP* + DMSO) and MD vs. CT (*dsCBP* + TSA). Genes with epifactor domains showed separately in a second excel sheet. Expression of Met and SRC in all three treatments showed in another excel sheet.

Supplementary Excel S3: Excel sheet containing details of 456 genes suppressed by CBP RNAi (MD vs. CD) and their relative expression in all other comparisons such as MD vs. CT, MD vs. MJ, MD vs. CJ and MD vs. MT.

Supplementary Excel S4: Excel sheet containing details of 181 genes responded after CBP RNAi and subsequent JH III treatment.

Supplementary Excel S5: Excel sheet containing details of 602 genes responded after CBP RNAi and subsequent TSA treatment.

Supplementary Excel S6: Excel sheet containing details of gene clusters obtained after Venn diagram analysis shown in main figure 6B.

Supplementary Table S1

Gene	Primer sequence(5'-3')	Direction
qRT-PCR primers		
TcasGA2_TC010839	CAGGGCCAGGAAATCAAG	Forward
	GCTGAAGGCGTTGTAGAC	Reverse
TcasGA2_TC000148	AACGAGCTCAACAATGGG	Forward
	CGCCGTATTTGTCCGAAA	Reverse
TcasGA2_TC012853	TCTGGGTCTATGACGGTAAG	Forward
	ATTCCTGGCCCATGTTTG	Reverse
GB16984	GGTGTTCAGTTCGGGTTC	Forward
	TCGATGAGTGTCGTAGGG	Reverse
TcasGA2_TC013193	ACTGGAAACCACGAAACG	Forward
	TAGCCGAAGTCAGGGAAA	Reverse
TcasGA2_TC007094	ATCGCGTGTGTGGAAATC	Forward
	TCGAAAGTGGTGCCAAAG	Reverse
TcasGA2_TC006721	TCACGCCTAAGCAGTTTG	Forward
	CGGGAACTTGGTGACTTATC	Reverse
TcasGA2_TC011641	GGACGGAGACAGACAAATC	Forward
	GTCGCTCGTCGAAATATCC	Reverse
TcasGA2_TC003157	CGGTCTTCACACACAGTTAG	Forward
	GATAAACCCACTTCCTGGTC	Reverse

Note: Primers for the rest of the genes such as 4EBP, CBP, TcasGA2_TC013402, Kr-h1, Rp49, Nuclear receptor coactivator 1(SRC), Methoprene-tolerant, dsCBP will be found in our earlier publications mentioned below:

1. Roy A, George S, Palli SR: Multiple functions of CREB-binding protein during postembryonic development: identification of target genes. BMC Genomics 2017, 18(1):996.
2. Jingjing Xu, Amit Roy & Subba Reddy Palli: CREB-binding protein plays key roles in juvenile hormone action in the red flour beetle, *Tribolium Castaneum*. Scientific Reports 2018, 8:1426

Supplementary Table S2

Sample	Total reads	Reads after QC*	% reads after QC	Mapped reads to Exon	% mapped	Uniquely mapped reads#
**MD1**	32,557,904	28,931,719	88.36	27,219,524	94.08	25,401,748
**MD2**	23,170,469	20,628,503	89.03	19,311,911	93.62	18,023,658
**MD3**	34,551,747	30,877,488	89.37	28,743,041	93.09	26,884,212
**MJ1**	20,396,843	18,501,186	90.71	17,210,535	93.02	16,035,311
**MJ2**	27,813,179	24,739,983	88.95	23,029,387	93.09	21,409,539
**MJ3**	15,842,254	14,225,539	89.79	13,338,160	93.76	12,450,612
**MT1**	33,863,136	23,336,656	68.91	21,159,900	90.60	19,738,084
**MT2**	25,746,558	21,219,162	82.42	19,009,018	89.58	17,686,189
**MT3**	26,406,181	10,372,230	39.28	8,661,657	83.51	8,123,452
**CD1**	32,579,815	29,284,406	89.89	27,312,718	93.27	25,754,030
**CD2**	32,777,122	28,905,897	88.19	27,790,577	92.68	25,257,369
**CD3**	33,049,791	27,857,657	84.29	26,227,606	94.15	24,742,314
**CJ1**	34,942,868	24,855,587	71.22	23,545,213	94.61	22,150,240
**CJ2**	26,406,828	21,384,097	80.98	20,146,622	94.21	18,937,081
**CJ3**	38,977,508	32,827,495	84.22	29,580,098	90.11	27,907,260
**CT1**	33,419,875	28,880,267	86.42	26,320,102	91.14	24,683,695
**CT2**	32,414,000	25,896,217	79.89	23,019,658	88.89	21,520,191
**CT3**	28,211,808	24,411,723	86.53	22,239,670	91.10	20,859,554

*QC= **Quality control # only reads uniquely mapped to Exon regions are considered for gene expression analysis.**

Supplementary Figure S1

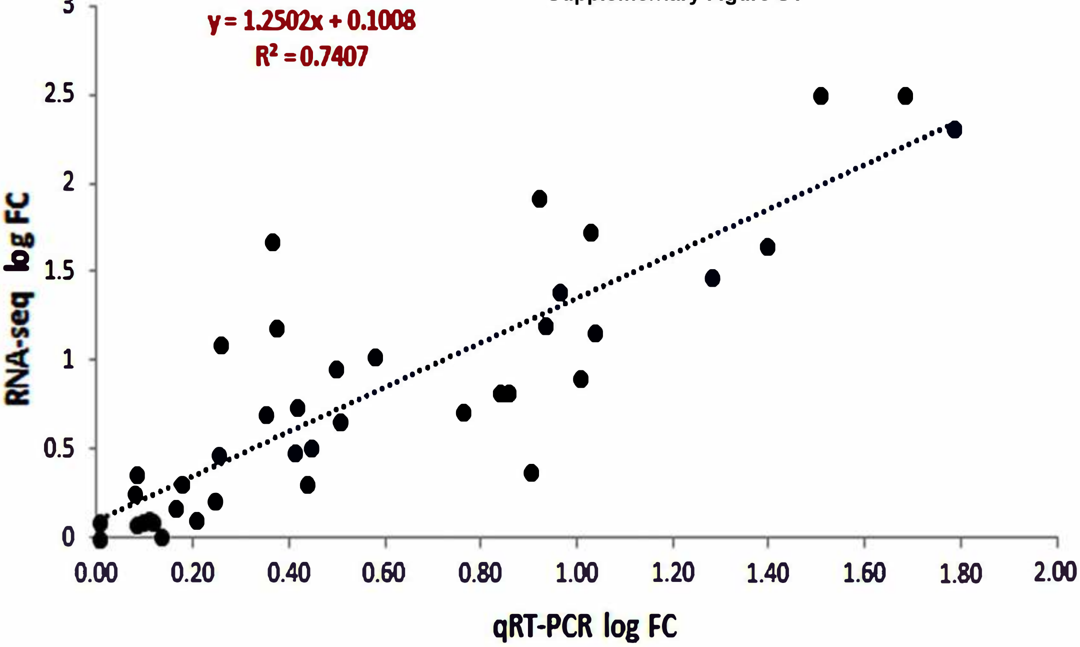

Supplementary Figure S2

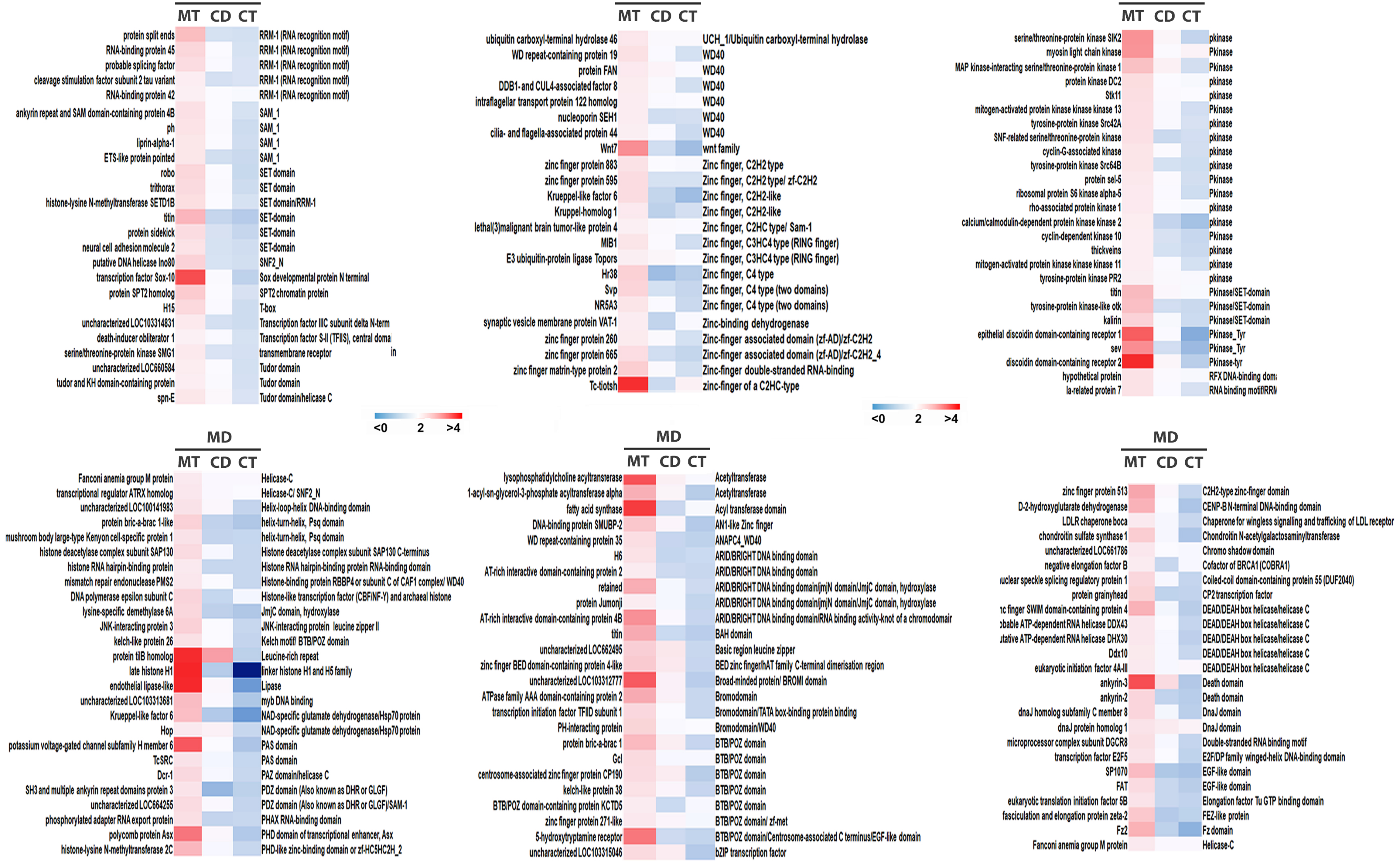

Supplementary Figure S3

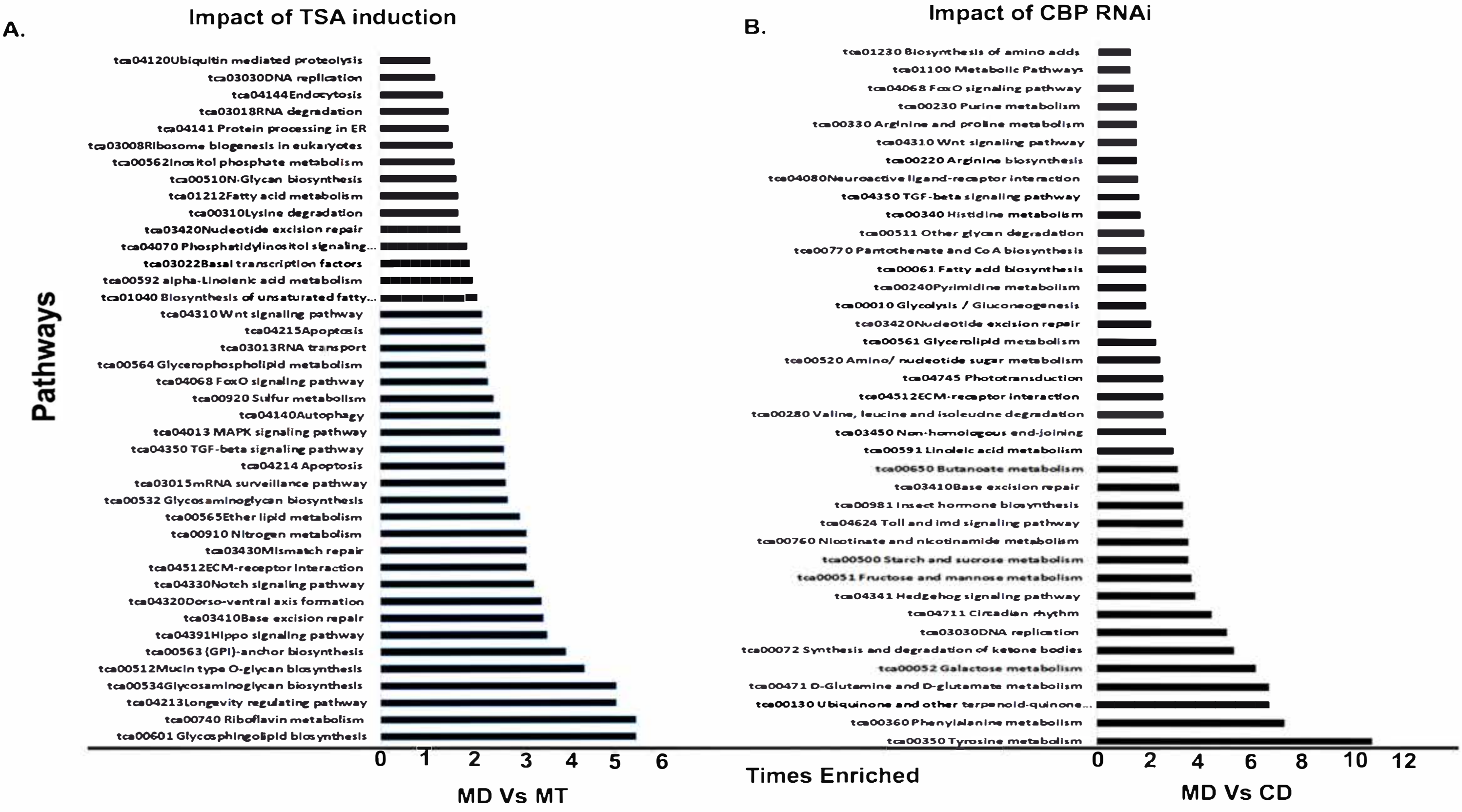

Supplementary Figure S4

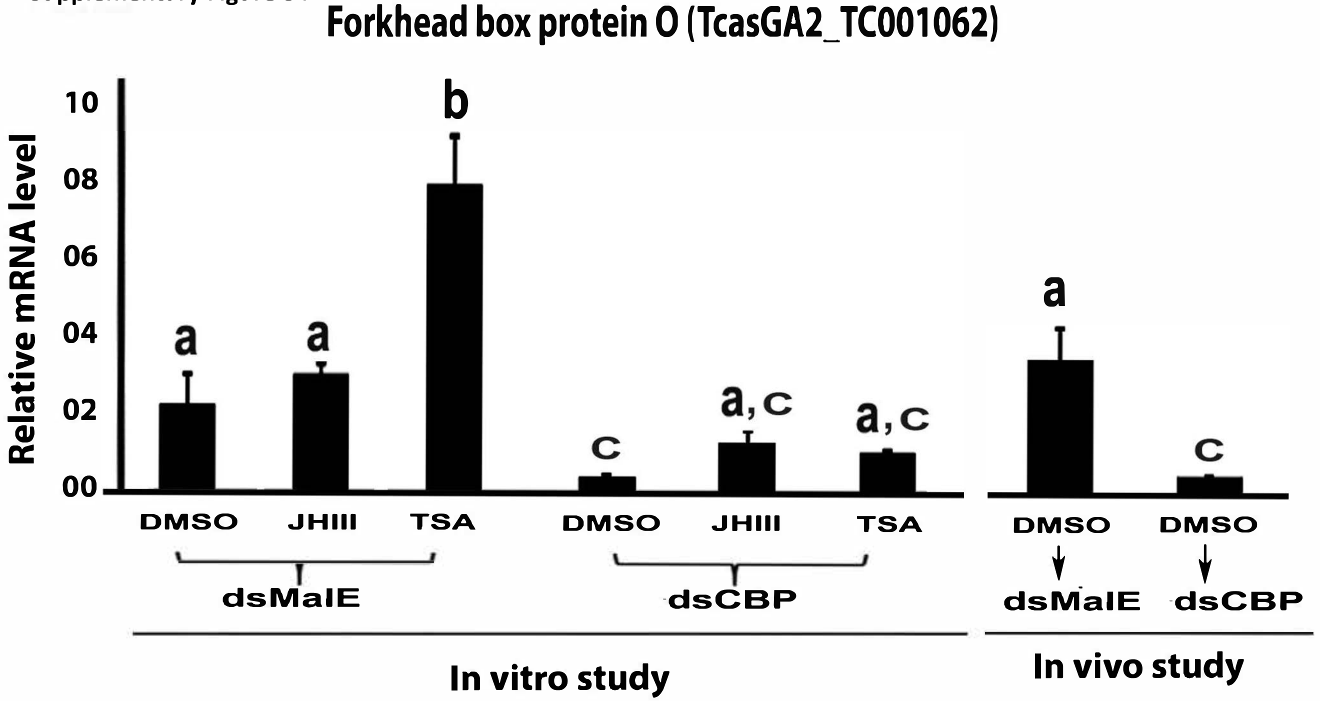

Supplement: Supplementary file 1 — Table S1. List of primers. Table S2. Detailed RNA seq statistics. Table is showing a summary of read statistics after Illumina Hi-seq 4000 sequencing for individual biological replicates in different treatments. Figure S1. Correlation of gene expression levels of selected genes (15) by comparing both RT-qPCR and RNA-seq data. Individual log fold changes obtained by RT-qPCR and RNA-seq for each gene in the sample group. Figure S2. Relative expression of the epi-factor domain-containing genes in MD vs. MT, MD vs. CD and MD vs. CT comparisons. Heatmaps are illustrating the relative expression of genes with the epi-factor domain, in all three treatments (MT, CD, and CT). Figure S3. Pathway enrichment analysis. A) Pathways enriched after TSA induction. B) Pathway affected due to CBP RNAi. Here, the negative impact is equivalent to the enrichment. Figure S4. Relative expression of FoxO transcription factor after different treatments in TcA cells and CBP RNAi in larvae. The data shown are the mean + S.E. (n = 4). (Letters represent significance at 95% CI). (PDF 9301 kb) [file 12864_2018_5323_MOESM1_ESM.pdf]
